# High precision compact numerical approximation in exponential form for the system of 2D quasilinear elliptic BVPs on a discrete irrational region

**DOI:** 10.1016/j.mex.2022.101790

**Published:** 2022-07-23

**Authors:** R.K. Mohanty, Nikita Setia, Gunjan Khurana, Geetan Manchanda

**Affiliations:** a Department of Mathematics, South Asian University, Akbar Bhawan, Chanakyapuri, New Delhi, 110021, India; b Department of Mathematics, Shaheed Bhagat Singh College, University of Delhi, New Delhi, 110017, India; c Department of Mathematics, I.P. College for Women, University of Delhi, Delhi, 110054, India; d Department of Mathematics, Maitreyi College, University of Delhi, Delhi, 110021, India

**Keywords:** Quasilinear elliptic partial differential equation, Fourth order numerical approximation, Exponential form, Irrational domain with unequal mesh, Convergence analysis, Burgers’ equation, Navier-Stokes equations

## Abstract

This article presents a new approximation of order four in exponential form for two-dimensional (2D) quasilinear partial differential equation (PDE) of elliptic form with solution domain being irrational. It is further extended for application to a system of quasilinear elliptic PDEs with Dirichlet boundary conditions (DBCs). The main highlights of the method framed in this article are as under:

• It uses a 9-point stencil with unequal mesh to approach the solution. The error analysis is discussed to authenticate the order of convergence of the proposed numerical approximation.
• Various validating problems, for instance the Burgers’ equation, Poisson equation in cylindrical coordinates, Navier-Stokes (NS) equations in rectangular and cylindrical coordinates are solved using the proposed techniques to depict their stability. The proposed approximation produces solution free of oscillations for large values of Reynolds Number in the vicinity of a singularity.
• The results of the proposed method are superior in comparison to the existing methods of [49] and [56].

## Graphical abstract

**Figure:**
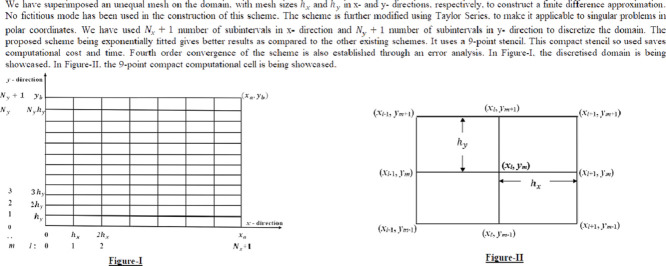

Specifications table

**Table:** 

Subject area	*Mathematics*
More specific subject area	*Partial Differential Equations*
Name of your method	*Finite Difference Method*
Name and reference of original method	• M.M. Chawla, A fourth-order tridiagonal finite difference method for general non-linear two-point boundary value problems with mixed boundary conditions, IMA Journal of Applied Mathematics. 21(1) (1978) 83–93. https://doi.org/10.1093/imamat/21.1.83
	• M.K. Jain, R.K. Jain and R.K. Mohanty, A fourth order difference method for elliptic equations with non-linear first derivative terms, Numerical Methods for Partial Differential Equation., 5 (1989) 87-95. https://doi.org/10.1002/num.1690050203
Resource availability	MATLAB

## Method details

### Scientific mathematical background

Consider the following equation

(1.1) $$a\left(x,y,u\right)u_{xx}+b\left(x,y,u\right)u_{yy}=\phi \left(x,y,u,u_{x},u_{y}\right).$$

Eq. (1.1) is a two-dimensional (2D) elliptic PDE (EPDE) in quasilinear form, with solution domain $\Omega_{h}=\left\{\left(x,y\right)\;|0<x<x_{a},\;0<y<y_{b}\right\}$ and boundary Γ, $x_{a}$ and $y_{b}$ being two real numbers on the positive *x*- and *y*-axis respectively, such that the fraction $\frac{x_{a}}{y_{b}}$ is irrational.

The Dirichlet values on the boundary Γ are

(1.2) $$u\left(x,y\right)=u_{0}\left(x,y\right),\left(x,y\right)\in \Gamma .$$

The following assumptions for EPDE (1.1) with respect to the boundary conditions (1.2) are made:

I. a(x,y,u).b(x,y,u)>0 in $\Omega_{h}$.
II. $u\left(x,y\right)\in C^{6}\left(\Omega_{h}\right)$ and $a\left(x,y,u\right),\;b\left(x,y,u\right)\in C^{4}\left(\Omega_{h}\right)$.
III. $(\frac{\partial \phi }{\partial u})\geq 0$ and $\phi \in C^{4}\left(\Omega_{h}\right)$.
IV. $|\frac{\partial \phi }{\partial u_{x}}|\leq G\equiv \;$a positive constant, $|\frac{\partial \phi }{\partial u_{y}}|\leq H\equiv \;$a positive constant.

The condition (I) is required to satisfy the elliptic condition, the condition (II) is required for a valid local truncation error. Conditions (III)-(IV) are the sufficient conditions for the convergence of the numerical solution (Jain et al. [1]). Further, we presume that the solution of the boundary value problem (BVP) (1.1)–(1.2) is unique and exists in the domain $\Omega_{h}$.

In the past, many high order compact numerical approximations for the nonlinear EPDEs have aroused renewed interest amongst the researchers and different type of techniques have been developed. A compact finite difference method (FDM) is restricted to cells surrounding any given grid and does not extend further, so it is convenient for computation since no special techniques are required for nodes near the boundary (Chawla [64]). Many problems of physical significance like the diffusion equation with first derivative convection terms, viscous Burgers’, and viscous Navier-Stokes (NS) equations in rectangular, cylindrical and spherical coordinates, are modeled by the EPDEs. Most of the numerical methods to solve EPDEs are iterative methods. We have attempted to solve numerically the coupled nonlinear equations like NS equations which show up in diverse areas of physics and mathematics. Li et al. [2] have expressed the NS equations in stream function-vorticity form and solved the system using nonlinear SOR iteration method. Yanwen et al. [3] have discretized the convection terms with high order upwind compact difference approximation and solved the incompressible NS equations in a rectangular domain. In 2009, Shah and Yuan [4] have proposed upwind compact approximations, of order three and five, for the solution of NS equations with artificial compressibility. These methods involved a flux-difference splitting technique. On the basis of the projection method, a high order FDM has been developed by Tian et al. [5]. This method is applied over the 2D incompressible NS equations in primitive variables. Using a fine grid mesh and a tri-diagonal solver, a new compact fourth order approximation has been employed by Erturk and Gökcöl [7] to solve the NS equations. Liu and Wang [8] have proposed a new high order FDM in terms of mean-vorticity formulation. The solutions obtained through this FDM were analyzed for the primitive equations of significant oceanic and atmospheric flow. A compact MAC FDM of high accuracy involving a staggered mesh has been developed by Ito and Qiao [9] for the solution of Stokes equations with Dirichlet boundary conditions (DBCs) on the velocity. A fourth-order compact nine-point 2D stencil has been formulated in [10], [11], [12] for the NS equations in steady stream function-vorticity form. A spatial approximation based on orthogonal spline collocation has been proposed by Fairweather et al. [13] for the 2D NS equations in fully coupled stream function-vorticity form. Various numerical approximations for 2D EPDEs with significant linear first derivative terms have been studied in [14], [15], [16]. In practical, numerical approximations for nonlinear EPDEs are of extreme interest in applied physics and applied mathematics. Different approximations have been developed for solving EPDEs, in particular finite element methods (FEMs), finite volume methods (FVMs) and FDMs [17], [18], [19], [20], [21], [22]. Ananthakrishnaiah et al. [23] have analyzed a fourth order approximation for 2D mildly nonlinear EPDEs. Different types of fourth order approximations for the solution for 2D quasilinear elliptic BVPs and their normal derivatives have been studied in [24], [25], [26]. Zhang [27] and Saldanha [28] have derived compact numerical approximations for 2D elliptic BVPs and discussed convergence and performances of iterative methods. Arabshahi and Dehghan [29] have adopted preconditioning techniques to solve linear EPDEs.

Let us now document some of the related research works done in the past one decade. Several meshless techniques for solving the various counterparts of Eq. (1.1) have been proposed by various authors. In 2013, Aziz et al. [59] have proposed two new efficient collocation methods to obtain the solution of a linear EPDE with a variety of boundary conditions. Their methods were based on Haar and Legendre Wavelets. This work has been further extended, in the year 2017, for applicability to nonlinear EPDEs with improved efficiency by Aziz and Islam [61]. In 2015, Oberman and Zwiers [30] have introduced a combination of adaptive and monotone FDMs to non-linear elliptic and parabolic PDEs with free boundaries. Subsequently, in 2017, a higher order method using the similar approach has been developed by Hamfeldt and Salvador [31]. A meshless technique for solving a 3D linear EPDE has been designed by Gavete et al. [32] in 2016, and for a 2D non-linear EPDE by Gavete et al. [33] in 2017. In 2019, Oruç [34] have solved the Poisson equation with mixed boundary conditions and irregular domain using a generalized FDM. In the same year, two new numerical techniques for two and three dimensional EPDEs with regular interfaces have been proposed by Haider et al. [63]. These techniques were based on meshless collocation and Haar wavelet collocation. In 2021, Milewski [35] have proposed a meshless technique for solving a 2D linear EPDE with Dirichlet and Neumann conditions. Apart from the meshless approaches, Mohanty and Setia [36] have designed a highly accurate compact half-step discretization for a system of general non-linear 2D EPDEs in the year 2013. A compact FDM of high order accuracy for a 2D Poisson equation has been given by Zhai et al. [37] in the following year. Further, in 2015, Papanikos and Gousidou-Koutita [39] have studied and compared the numerical results obtained upon application of the central difference scheme and an FEM algorithm over a general linear second order EPDE. The same year, Islam et al. [60] reported a new numerical technique based on Haar Wavelet collocation and a meshless method involving different types of radial basis functions for a 2D Poisson equation with a two-point non-local boundary condition and an integral boundary condition. In 2016, Mittal et al. [41] have given a class of FDMs for solving 1D and 2D elliptic and parabolic equations. The accuracy of this scheme is atleast two. It is successfully applicable to time-dependent NS equations in cylindrical polar coordinates. RBF generated FDMs, successfully applicable to the NS equations in cylindrical polar coordinates, have also been proposed by Bayona et al. [40] in the year 2017. This is. Afterwards, in 2018, Mittal and Ray [42] have proposed a new second order FDM for 2D and 3D non-linear EPDEs. The EPDEs involved in [41] and [42] have discontinuous coefficients and singular source terms. Raeli et al. [45] have developed an FDM for the variable coefficient Poisson equation. Aziz *et al*. [62] have designed a new collocation method for three dimensional nonlinear EPDEs with DBCs. This method is based on Haar Wavelets. A second order FDM for a system of EPDEs in a square domain has been given by Pandey and Jaboob [48]. Zhang et al. [6] have proposed a highly accurate discontinuous Galerkin method for solving the 2D NS equations in conservative form on arbitrary grids. In 2020, Li and Zhang [50] have reduced the classical continuous FEM to a high order FDM applied to a 2D elliptic equation with smooth coefficients on a rectangular domain. A new bi-cubic spline collocation method of fourth order accuracy has been designed by Singh and Singh [57] for a linear EPDE with DBCs. Most recently, new high accuracy approximations in exponential form for solving two-point BVPs on a variable mesh, and 2D nonlinear EPDEs on an unequal uniform mesh have been reported in [51], [52], [53], [54], [55]. Mohanty and Kumar [56], and Priyadarshini and Mohanty [47,49] have proposed new high accuracy numerical algorithms for 2D quasilinear EPDEs using unequal mesh. Singh et al. [58] have developed a collocation method-based technique for second order linear EPDEs with irregularities in one and two dimensions.

**Fig. 1:**
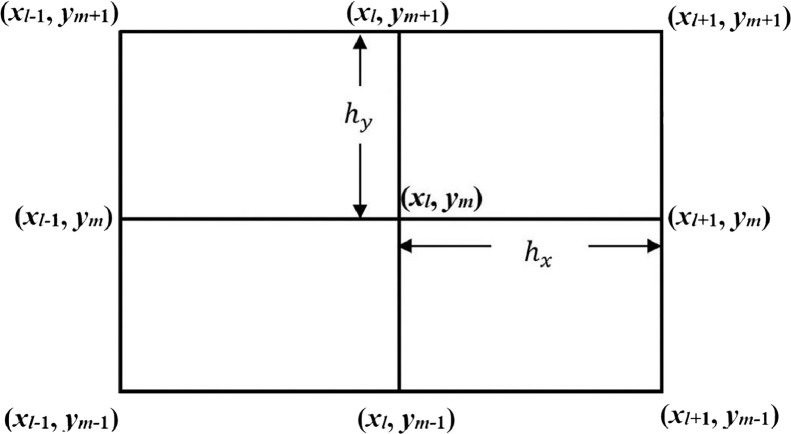
Nine-point stencil with unequal mesh sizes in x- and y- directions.

In the proposed domain $\Omega_{h}$, the grid sizes in both *x*- and *y-*directions cannot be equal. It is necessary to develop an appropriate stable compact highly accurate computational method for the EPDE (1.1) using an unequal mesh. In this piece of work, we design a new compact numerical method in exponential form of order four on the given irrational domain for the solution of quasilinear elliptic PDE (1.1). We use nine-point stencil in the formulation of the method (Fig. 1). The outline of the rest of the paper is as follows: In Section 1.2, we frame the FDM in the exponential form on an irrational domain using unequal grid sizes in x- and y- directions. Further, in Section 1.3, we deduce the compact numerical approximation. The obtained discretization is extended for applicability to a system of 2D EPDEs with DBCs. A proof of convergence of the numerical method is given in Section 1.4. The error is proved to be of fourth-order accuracy. Section 2 comprises of some numerical illustrations for method validation. Different kinds of benchmark elliptic BVPs are included for numerical illustrations to demonstrate the fourth order accuracy and application of the method discussed.

### Discretization procedure

The 2D nonlinear EPDE is given by

(2) $$a\left(x,y\right)u_{xx}+b\left(x,y\right)u_{yy}=\phi \left(x,y,u,u_{x},u_{y}\right),0<x<x_{a},\;0<y<y_{b}.$$

The DBCs for (2) are given by the Eq. (1.2). The discretization is implemented on the solution domain $\Omega_{h}$. Let $h_{x}>0$ and $h_{y}>0$ be the mesh sizes in *x*- and y- directions, respectively, where $h_{x}\neq h_{y}.$ The coordinate points of the mesh so generated are $\left(x_{l},y_{m}\right)$, where $x_{l}=lh_{x}$ and $y_{m}=mh_{y}$, for $l=0,1,\ldots ,(N_{x}+1)$ and $m=0,1,\ldots ,(N_{y}+1)$, such that ($N_{x}+1)h_{x}=x_{a}$ and $\left(N_{y}+1\right)h_{y}=y_{b}.$ At each mesh point $\left(x_{l},y_{m}\right)$, the exact solution value is denoted by $U_{l,m}$, and approximate solution value is denoted by $u_{l,m}$. Further, let $a_{l,m}$ denote $a(x_{l},y_{m})$ and $b_{l,m}$ denote $b(x_{l},y_{m})$.

For every mesh point $\left(x_{l},y_{m}\right)$ and for *S* = *a* and *b*, we define

(3) $$S_{pq}=\frac{\partial^{p+q}S}{\partial x^{p}\partial y^{q}},\;\text{for}p,q=0,1,2,$$

Let $p_{1}=\frac{-h_{x}a_{10}}{6a_{00}}$ and $p_{2}=\frac{-h_{y}b_{01}}{6b_{00}}$.

Further, for each $\left(x_{l},y_{m}\right)$, we denote

(4) $$\begin{array}{c} U_{0}=U_{l,m},\;U_{1}=U_{l+1,m},\;U_{2}=U_{l-1,m},\;U_{3}=U_{l,m+1},\;U_{4}=U_{l,m-1},\\ U_{5}=U_{l+1,m+1},\;U_{6}=U_{l+1,m-1},\;\;U_{7}=U_{l-1,m+1},\;U_{8}=U_{l-1,m-1} \end{array}$$

Using (4), we generate an approximation using unequal grid sizes in x- and y- directions by defining the following

(5.1) $${\hat{U}}_{x\;l,m}=\frac{1}{2h_{x}}\left(U_{1}-U_{2}\right),$$

(5.2) $${\hat{U}}_{x\;l+1,m}=\frac{1}{2h_{x}}\left(3U_{1}-4U_{0}+U_{2}\right),$$

(5.3) $${\hat{U}}_{xl-1,m}=\frac{1}{2h_{x}}\left(-3U_{2}+4U_{0}-U_{1}\right),$$

(5.4) $${\hat{U}}_{x\;l,m+1}=\frac{1}{2h_{x}}\left(U_{5}-U_{7}\right),$$

(5.5) $${\hat{U}}_{x\;l,m-1}=\frac{1}{2h_{x}}\left(U_{6}-U_{8}\right),$$

(5.6) $${\hat{U}}_{y\;l,m}=\frac{1}{2h_{y}}\left(U_{3}-U_{4}\right),$$

(5.7) $${\hat{U}}_{y\;l+1,m}=\frac{1}{2h_{y}}\left(U_{5}-U_{6}\right),$$

(5.8) $${\hat{U}}_{y\;l-1,m}=\frac{1}{2h_{y}}\left(U_{7}-U_{8}\right),$$

(5.9) $${\hat{U}}_{y\;l,m+1}=\frac{1}{2h_{y}}\left(3U_{3}-4U_{0}+U_{4}\right),$$

(5.10) $${\hat{U}}_{y\;l,m-1}=\frac{1}{2h_{y}}\left(-3U_{4}+4U_{0}-U_{3}\right),$$

(5.11) $${\hat{U}}_{xx\;l,m}=\frac{1}{h_{x}^{2}}\left(U_{1}-2U_{0}+U_{2}\right),$$

(5.12) $${\hat{U}}_{yy\;l,m}=\frac{1}{h_{y}^{2}}\left(U_{3}-2U_{0}+U_{4}\right),$$

(5.13) $${\hat{U}}_{xx\;l,m+1}=\frac{1}{h_{x}^{2}}\left(U_{5}-2U_{3}+U_{7}\right),$$

(5.14) $${\hat{U}}_{xx\;l,m-1}=\frac{1}{h_{x}^{2}}\left(U_{6}-2U_{4}+U_{8}\right),$$

(5.15) $${\hat{U}}_{yy\;l+1,m}=\frac{1}{h_{y}^{2}}\left(U_{5}-2U_{1}+U_{6}\right),$$

(5.16) $${\hat{U}}_{yy\;l-1,m}=\frac{1}{h_{y}^{2}}\left(U_{7}-2U_{2}+U_{8}\right),$$

(5.17) $${\hat{U}}_{xxy\;l,m}=\frac{1}{2h_{x}^{2}h_{y}}\left[U_{5}-U_{6}+U_{7}-U_{8}-2\left(U_{3}-U_{4}\right)\right],$$

(5.18) $${\hat{U}}_{xyy\;l,m}=\frac{1}{2h_{y}^{2}h_{x}}\left[U_{5}+U_{6}-U_{7}-U_{8}-2\left(U_{1}-U_{2}\right)\right],$$

(5.19) $${\hat{U}}_{xxyy\;l,m}=\frac{1}{h_{x}^{2}h_{y}^{2}}\left[U_{5}+U_{6}+U_{7}+U_{8}-2\left(U_{1}+U_{2}+U_{3}+U_{4}\right)+4U_{0}\right],$$

(5.20) $${\hat{\Phi }}_{l\pm 1,m}=\phi \left(x_{l\pm 1},y_{m},U_{l\pm 1,m},{\hat{U}}_{x\;l\pm 1,m},{\hat{U}}_{y\;l\pm 1,m}\right),$$

(5.21) $${\hat{\Phi }}_{l,m\pm 1}=\phi \left(x_{l},y_{m\pm 1},U_{l,m\pm 1},{\hat{U}}_{x\;l,m\pm 1},{\hat{U}}_{y\;l,m\pm 1}\right),$$

(5.22) $$\begin{array}{ccc} {\hat{\hat{U}}}_{x\;l,m} & = & {\hat{U}}_{x\;l,m}-\frac{h_{x}}{12a_{00}}\left({\hat{\Phi }}_{l+1,m}-{\hat{\Phi }}_{l-1,m}\right)+\frac{h_{x}b_{00}}{12a_{00}}\left({\hat{\;U}}_{yy\;l+1,m}-{\hat{\;U}}_{yy\;l-1,m}\right)\\ & & +\;\frac{h_{x}^{2}}{6a_{00}}\left(a_{10}{\hat{\;U}}_{xx\;l,m}+b_{10}{\hat{\;U}}_{yy\;l,m}\right), \end{array}$$

(5.23) $$\begin{array}{ccc} {\hat{\hat{U}}}_{y\;l,m} & = & {\hat{U}}_{y\;l,m}-\frac{h_{y}}{12b_{00}}\left({\hat{\Phi }}_{l,m+1}-{\hat{\Phi }}_{l,m-1}\right)+\frac{h_{y}a_{00}}{12b_{00}}\left({\hat{\;U}}_{xx\;l,m+1}-{\hat{\;U}}_{xx\;l,m-1}\right)\\ & & +\;\frac{h_{y}^{2}}{6b_{00}}\left(a_{01}{\hat{\;U}}_{xx\;l,m}+b_{01}{\hat{\;U}}_{yy\;l,m}\right), \end{array}$$

(5.24) $$\begin{array}{ccc} {\tilde{\tilde{U}}}_{x\;l,m} & = & {\hat{U}}_{x\;l,m}-\frac{h_{x}}{8a_{00}}\left({\hat{\Phi }}_{l+1,m}-{\hat{\Phi }}_{l-1,m}\right)+\frac{h_{x}b_{00}}{8a_{00}}\left({\hat{\;U}}_{yy\;l+1,m}-{\hat{\;U}}_{yy\;l-1,m}\right)\\ & & +\;\frac{h_{x}^{2}}{4a_{00}}\left(a_{10}{\hat{\;U}}_{xx\;l,m}+b_{10}{\hat{\;U}}_{yy\;l,m}\right), \end{array}$$

(5.25) $$\begin{array}{ccc} {\tilde{\tilde{U}}}_{y\;l,m} & = & {\hat{U}}_{y\;l,m}-\frac{h_{y}}{8b_{00}}\left({\hat{\Phi }}_{l,m+1}-{\hat{\Phi }}_{l,m-1}\right)+\frac{h_{y}a_{00}}{8b_{00}}\left({\hat{\;U}}_{xx\;l,m+1}-{\hat{\;U}}_{xx\;l,m-1}\right)\\ & & +\;\frac{h_{y}^{2}}{4b_{00}}\left(a_{01}{\hat{\;U}}_{xx\;l,m}+b_{01}{\hat{\;U}}_{yy\;l,m}\right), \end{array}$$

(5.26) $${\hat{\hat{\Phi }}}_{\;l,m}=\phi \left(x_{l},y_{m},U_{l,m},{\hat{\hat{U}}}_{x\;l,m},{\hat{\hat{U}}}_{y\;l,m}\right),$$

(5.27) $${\tilde{\tilde{\Phi }}}_{\;l,m}=\phi \left(x_{l},y_{m},U_{l,m},{\tilde{\tilde{U}}}_{x\;l,m},{\tilde{\tilde{U}}}_{y\;l,m}\right).$$

Then, at every mesh point $\left(x_{l},y_{m}\right)$ of the irrational domain $\Omega_{h}$, the proposed discretization of the EPDE (2) is given by

(6) $$\begin{array}{ccc} L_{u} & \equiv & \left[a_{00}+p_{1}h_{x}a_{10}+p_{2}h_{y}a_{01}+\frac{h_{x}^{2}}{12}a_{20}+\frac{h_{y}^{2}}{12}a_{02}\right]{\hat{U}}_{xxl,m}\\ & & +\;\left[b_{00}+p_{1}h_{x}b_{10}+p_{2}h_{y}b_{01}+\frac{h_{x}^{2}}{12}b_{20}+\frac{h_{y}^{2}}{12}b_{02}\right]{\hat{U}}_{yyl,m}\\ & & +\;\left[p_{2}h_{y}a_{00}+\frac{h_{y}^{2}}{6}a_{01}\right]{\hat{U}}_{xxyl,m}+\left[p_{1}h_{x}b_{00}+\frac{h_{x}^{2}}{6}b_{10}\right]{\hat{U}}_{xyyl,m}\\ & & +\;\frac{1}{12}\left[h_{x}^{2}b_{00}+h_{y}^{2}a_{00}\right]{\hat{U}}_{xxyyl,m}\\ & = & {\hat{\Phi }}_{\;l,m}\exp\left[\frac{\left(1+6p_{1}\right){\hat{\Phi }}_{l+1,m}+\left(1-6p_{1}\right){\hat{\Phi }}_{l-1,m}+\left(1+6p_{2}\right){\hat{\Phi }}_{l,m+1}+\left(1-6p_{2}\right){\hat{\Phi }}_{l,m-1}-4{\tilde{\Phi }}_{l,m}}{12{\hat{\Phi }}_{l,m}}\right]\\ & & +\;{\hat{T}}_{l,m}, \end{array}$$

where ${\hat{T}}_{l,m}=O\left(h_{x}^{4}+h_{x}^{2}h_{y}^{2}+h_{y}^{4}\right)$is the local truncation error (LTE).

Incorporating the prescribed Dirichlet values from the boundary, the approximation (6) may be expressed as a block tri-diagonal matrix, that may be iteratively dealt with for a solution by choosing an appropriate block- iterative method ([38,43,44,46]).

### Derivation of the algorithms in the exponential form

At any mesh point $\left(x_{l},y_{m}\right)$, the EPDE (2) can be expressed as

(7) $$\;a_{00}U_{20}+b_{00}U_{02}=\phi \left(x_{l},y_{m},U_{l,m},U_{x\;l,m\;},\;U_{y\;l,m}\right)=\Phi_{l,m}.$$

From (7), it is simple to observe that

(8) $$\begin{array}{ccc} L_{u} & \equiv & \left[a_{00}+p_{1}h_{x}a_{10}+p_{2}h_{y}a_{01}+\frac{h_{x}^{2}}{12}a_{20}+\frac{h_{y}^{2}}{12}a_{02}\right]{\hat{U}}_{xx\;l,m}\\ & & +\;\left[b_{00}+p_{1}h_{x}b_{10}+p_{2}h_{y}b_{01}+\frac{h_{x}^{2}}{12}b_{20}+\frac{h_{y}^{2}}{12}b_{02}\right]{\hat{U}}_{yyl,m}\\ & & +\;\left[p_{2}h_{y}a_{00}+\frac{h_{y}^{2}}{6}a_{01}\right]{\hat{U}}_{xxyl,m}+\left[p_{1}h_{x}b_{00}+\frac{h_{x}^{2}}{6}b_{10}\right]{\hat{U}}_{xyyl,m}\\ & & +\;\frac{1}{12}\left[h_{x}^{2}b_{00}+h_{y}^{2}a_{00}\right]{\hat{U}}_{xxyyl,m}\\ & = & \Phi_{l,m}\exp\left[\frac{\left(1+6p_{1}\right)\Phi_{\;l+1,m}+\left(1-6p_{1}\right)\Phi_{\;l-1,m}+\left(1+6p_{2}\right)\Phi_{\;l,m+1}+\left(1-6p_{2}\right)\Phi_{\;l,m-1}-4\Phi_{l,m}}{12\Phi_{l,m}}\right]\\ & & +\;O\left(h_{y}^{4}+h_{x}^{2}h_{y}^{2}+h_{y}^{2}\right). \end{array}$$

Simplifying the approximations (5.1)-(5.19), we obtain

(9.1) $${\hat{U}}_{x\;l,m}=U_{x\;l,m}+\frac{h_{x}^{2}}{6}U_{xxx\;l,m}+O\left(h_{x}^{4}\right),$$

(9.2) $${\hat{U}}_{x\;l\pm 1,m}=U_{x\;l\pm 1,m}-\frac{h_{x}^{2}}{3}U_{xxx\;l,m}\pm O\left(h_{x}^{3}\right),$$

(9.3) $${\hat{U}}_{x\;l,m\pm 1}=\;U_{x\;l,m\pm 1}+\frac{h_{x}^{2}}{6}U_{xxx\;l,m}\pm O\left(h_{x}^{2}h_{y}\right),$$

(9.4) $${\hat{U}}_{y\;l,m}=\;U_{y\;l,m}+\frac{h_{y}^{2}}{6}U_{yyy\;l,m}+O\left(h_{y}^{4}\right),$$

(9.5) $${\hat{U}}_{y\;l\pm 1,m}=\;U_{y\;l\pm 1,m}+\frac{h_{y}^{2}}{6}U_{yyy\;l,m}\pm O\left(h_{x}h_{y}^{2}\right),$$

(9.6) $${\hat{U}}_{y\;l,m\pm 1}=U_{y\;l,m\pm 1}-\frac{h_{y}^{2}}{3}U_{yyy\;l,m}\pm O\left(h_{y}^{3}\right),$$

(9.7) $${\hat{U}}_{xx\;l,m}=U_{xx\;l,m}+\frac{h_{x}^{2}}{12}U_{xxxx\;l,m}+O\left(h_{x}^{4}\right),$$

(9.8) $${\hat{U}}_{yy\;l,m}=\;U_{yy\;l,m}+\frac{h_{y}^{2}}{12}U_{yyyy\;l,m}+O\left(h_{y}^{4}\right),$$

(9.9) $${\hat{U}}_{xx\;l,m\pm 1}=\;U_{xx\;l,m\pm 1}\;+\frac{h_{x}^{2}}{12}U_{xxxx\;l,m}\pm O\left(h_{x}^{2}h_{y}\right),$$

(9.10) $${\hat{U}}_{yy\;l\pm 1,m}=U_{yy\;l\pm 1,m}+\frac{h_{y}^{2}}{12}U_{yyyy\;l,m}\pm O\left(h_{x}h_{y}^{2}\right),$$

(9.11) $${\hat{U}}_{xxy\;l,m}=U_{xxy\;l,m}+\;O\left(h_{x}^{2}+h_{y}^{2}\right),$$

(9.12) $${\hat{U}}_{xyy\;l,m}=U_{xyy\;l,m}+\;O\left(h_{x}^{2}+h_{y}^{2}\right),$$

(9.13) $${\hat{U}}_{xxyy\;l,m}=\;U_{xxyy\;l,m}+\;O\left(h_{x}^{2}+h_{y}^{2}\right).$$

Employing the approximations (9.2), (9.3), (9.5) and (9.6) in (5.20) and (5.21), and simplifying, we get

(10.1) $$\begin{array}{ccc} {\hat{\Phi }}_{l\pm 1,m} & = & \Phi_{l\pm 1,m}-\frac{h_{x}^{2}}{3}\;U_{xxx\;l,m}\;\phi_{u_{x}\;l,m}+\frac{h_{y}^{2}}{6}U_{yyy\;l,m}\phi_{u_{y}\;l,m}\\ & & +\;O\left(\pm h_{x}^{3}\pm h_{x}h_{y}^{2}+h_{x}^{4}+h_{x}^{2}h_{y}^{2}\right), \end{array}$$

(10.2) $$\begin{array}{ccc} {\hat{\Phi }}_{l,m\pm 1} & = & \Phi_{l,m\pm 1}+\frac{h_{x}^{2}}{6}U_{xxx\;l,m}\;\phi_{u_{x}\;l,m}-\frac{h_{y}^{2}}{3}U_{yyy\;l,m}\phi_{u_{y}\;l,m}\\ & & +\;O\left(\pm h_{y}^{3}\pm h_{y}h_{x}^{2}+h_{y}^{4}+h_{x}^{2}h_{y}^{2}\right). \end{array}$$

For higher order approximations of the derivatives, let us define

(11.1) $$\begin{array}{ccc} {\hat{\hat{U}}}_{x\;l,m} & = & {\hat{U}}_{x\;l,m}+\alpha_{11}h_{x}\left({\hat{\Phi }}_{l+1,m}-{\hat{\Phi }}_{l-1,m}\right)+\alpha_{12}h_{x}\left({\hat{\;U}}_{yy\;l+1,m}-{\hat{\;U}}_{yy\;l-1,m}\right)\\ & & +\;2h_{x}^{2}\left(\alpha_{13}{\hat{\;U}}_{xx\;l,m}+\alpha_{14}{\hat{\;U}}_{yy\;l,m}\right), \end{array}$$

(11.2) $$\begin{array}{ccc} {\hat{\hat{U}}}_{y\;l,m} & = & {\hat{U}}_{y\;l,m}+\beta_{11}h_{y}\left({\hat{\Phi }}_{l,m+1}-{\hat{\Phi }}_{l,m-1}\right)+\beta_{12}h_{y}\left({\hat{\;U}}_{xx\;l,m+1}-{\hat{\;U}}_{xx\;l,m-1}\right)\\ & & +\;2h_{y}^{2}\left(\beta_{13}{\hat{\;U}}_{xx\;l,m}+\beta_{14}{\hat{\;U}}_{yy\;l,m}\right), \end{array}$$

where $\alpha_{1j},\;\beta_{1j},$ *j* = 1,2,3,4, are the parameters to be estimated. Using the approximations (9.7)–(9.10), (10.1) and (10.2), in the expressions (11.1) and (11.2), and simplifying, we get

(12.1) $${\hat{\hat{U}}}_{x\;l,m}=U_{x\;l,m}+\frac{h_{x}^{2}}{6}T_{1}+O\left(h_{x}^{4}+h_{x}^{2}h_{y}^{2}\right),$$

(12.2) $${\hat{\hat{U}}}_{y\;l,m}=U_{y\;l,m}+\frac{h_{y}^{2}}{6}T_{2}+O\left(h_{y}^{4}+h_{x}^{2}h_{y}^{2}\right),$$

where

$$\begin{array}{ccc} T_{1} & = & \left(1+12\alpha_{11}a_{00}\right)U_{xxx\;l,m}+12\left(\alpha_{11}b_{00}+\alpha_{12}\right)U_{xyy\;l,m}\\ & & +\;12\left(\alpha_{11}a_{10}+\alpha_{13}\right)U_{xx\;l,m}+12\left(\alpha_{11}b_{10}+\alpha_{14}\right)U_{yy\;l,m},\\ T_{2} & = & \left(1+12\beta_{11}b_{00}\right)U_{yyy\;l,m}+12\left(\beta_{11}a_{00}+\beta_{12}\right)U_{xxy\;l,m}\\ & & +\;12\left(\beta_{11}a_{01}+\beta_{13}\right)U_{xx\;l,m}+12\left(\beta_{11}b_{01}+\beta_{14}\right)U_{yy\;l,m}. \end{array}$$

From (12.1)–(12.2), it is evident that for $T_{1}=T_{2}$=0, that is, for

$$\alpha_{11}=\frac{-1}{12a_{00}},\alpha_{12}=\frac{b_{00}}{12a_{00}},\alpha_{13}=\frac{a_{10}}{12a_{00}},\alpha_{14}=\frac{b_{10}}{12a_{00}},$$

$$\beta_{11}=\frac{-1}{12b_{00}},\beta_{12}=\frac{a_{00}}{12b_{00}},\beta_{13}=\frac{a_{01}}{12b_{00}},\beta_{14}=\frac{b_{01}}{12b_{00}},$$

we have

(13.1) $${\hat{\hat{U}}}_{x\;l,m}=U_{x\;l,m}+O\left(h_{x}^{4}+h_{x}^{2}h_{y}^{2}\right),$$

(13.2) $${\hat{\hat{U}}}_{y\;l,m}=U_{y\;l,m}+O\left(h_{y}^{4}+h_{x}^{2}h_{y}^{2}\right).$$

Thus, with the aid of (13.1)–(13.2), (5.26) simplifies to

(14) $${\hat{\hat{\Phi }}}_{\;l,m}=\Phi_{l,m}+O\left(h_{x}^{4}+h_{x}^{2}h_{y}^{2}+h_{y}^{4}\right).$$

Further, in order to enhance the accuracy of the designed FDM, let

(15.1) $$\begin{array}{ccc} {\tilde{\tilde{U}}}_{x\;l,m} & = & {\hat{U}}_{x\;l,m}+\alpha_{21}h_{x}\left({\hat{\Phi }}_{l+1,m}-{\hat{\Phi }}_{l-1,m}\right)+\alpha_{22}h_{x}\left({\hat{\;U}}_{yy\;l+1,m}-{\hat{\;U}}_{yy\;l-1,m}\right)\\ & & +\;2h_{x}^{2}\left(\alpha_{23}{\hat{\;U}}_{xx\;l,m}+\alpha_{24}{\hat{\;U}}_{yy\;l,m}\right), \end{array}$$

(15.2) $$\begin{array}{ccc} {\tilde{\tilde{U}}}_{y\;l,m} & = & {\hat{U}}_{y\;l,m}+\beta_{21}h_{y}\left({\hat{\Phi }}_{l,m+1}-{\hat{\Phi }}_{l,m-1}\right)+\beta_{22}h_{y}\left({\hat{\;U}}_{xx\;l,m+1}-{\hat{\;U}}_{xx\;l,m-1}\right)\\ & & +\;2h_{y}^{2}\left(\beta_{23}{\hat{\;U}}_{xx\;l,m}+\beta_{24}{\hat{\;U}}_{yy\;l,m}\right), \end{array}$$

where $\alpha_{2j},\;\beta_{2j},$ *j* = 1,2,3,4, are arbitrary parameters to be estimated. With the aid of (9.7)–(9.10), (10.1) and (10.2), in the approximations (15.1) and (15.2), and simplifying, we get

(16.1) $${\tilde{\tilde{U}}}_{x\;l,m}=U_{x\;l,m}+\frac{h_{x}^{2}}{6}T_{3}+O\left(h_{x}^{4}+h_{x}^{2}h_{y}^{2}\right),$$

(16.2) $${\tilde{\tilde{U}}}_{y\;l,m}=U_{y\;l,m}+\frac{h_{y}^{2}}{6}T_{4}+O\left(h_{y}^{4}+h_{x}^{2}h_{y}^{2}\right),$$

where

$$\begin{array}{ccc} T_{3} & = & \left(1+12\alpha_{21}a_{00}\right)U_{xxx\;l,m}+12\left(\alpha_{21}b_{00}+\alpha_{22}\right)U_{xyy\;l,m}\\ & & +\;12\left(\alpha_{21}a_{10}+\alpha_{23}\right)U_{xx\;l,m}+12\left(\alpha_{21}b_{10}+\alpha_{24}\right)U_{yy\;l,m},\\ T_{4} & = & \left(1+12\beta_{21}b_{00}\right)U_{yyy\;l,m}+12\left(\beta_{21}a_{00}+\beta_{22}\right)U_{xxy\;l,m}\\ & & +\;12\left(\beta_{21}a_{01}+\beta_{23}\right)U_{xx\;l,m}+12\left(\beta_{21}b_{01}+\beta_{24}\right)U_{yy\;l,m} \end{array}$$

As a result of (16.1)–(16.2), from (5.27), we have

(17) $${\tilde{\tilde{\Phi }}}_{l,m}=\Phi_{l,m}+\frac{h_{x}^{2}}{6}\;T_{3}\;\phi_{u_{x}l,m}+\frac{h_{y}^{2}}{6}T_{4}\;\phi_{u_{y}l,m}+O\left(h_{x}^{4}+h_{x}^{2}h_{y}^{2}+h_{y}^{4}\right),$$

Finally, with the support of (10.1), (10.2), (14) and (17), from (6) and (8), the LTE can be computed as

(18) $${\hat{T}}_{l,m}=\;-\frac{h_{x}^{2}}{3}\left(2T_{3}+U_{xxx\;l,m}\right){\phi_{u_{x}}}_{{}_{l,m}}-\frac{h_{y}^{2}}{3}\left(2T_{4}+U_{yyy\;l,m}\right){\phi_{u_{y}}}_{{}_{l,m}}+O\left(h_{x}^{4}+h_{x}^{2}h_{y}^{2}+h_{y}^{4}\right).$$

Since the LTE ${\hat{T}}_{l,m}$ is required to be of $O(h_{x}^{4}+h_{x}^{2}h_{y}^{2}+h_{y}^{4})$, the coefficients of $h_{x}^{2}$ and $h_{y}^{2}$ in (18) should vanish, hence the parameters attain the following values

$$\begin{array}{ccc} & & \left(\alpha_{21},\alpha_{22},\alpha_{23},\;\alpha_{24}\right)\\ & & =\;\frac{1}{8a_{00}}\left(-1,b_{00},\;a_{10},b_{10}\right),\\ & & \left(\beta_{21},\beta_{22},\;\beta_{23},\;\beta_{24}\right)\;\\ & & =\;\frac{1}{8b_{00}}\left(-1,a_{00},\;a_{01},b_{01}\right), \end{array}$$

and the LTE (18) is reduced to ${\hat{T}}_{l,m}=\;O\left(h_{x}^{4}+h_{x}^{2}h_{y}^{2}+h_{y}^{4}\right)$.

The technique discussed in [49] can be used to obtain numerical approximation of order four for the quasilinear EPDE (1.1). Further, the proposed method (6) for the single equation can be stretched out to the system of quasilinear EPDE in vector-matrix form, as discussed in [36].

**Note: Application to elliptic BVP in r-z plane**

Let us now discuss the implementation of the proposed discretization to the following EPDE in r-z plane

(19) $$u_{rr}+\frac{1}{r}u_{r}+u_{zz}=g\left(r,z\right),0<r<1,0<z<b.$$

Let $\delta_{r}U_{l}=U_{l+\frac{1}{2}}-U_{l-\frac{1}{2}},\mu_{r}U_{l}=\frac{1}{2}\left(U_{l+\frac{1}{2}}+U_{l-\frac{1}{2}}\right)$, and $h_{r}>0,\;h_{z}>0$ be the mesh sizes in radial and *z*-axis directions, respectively. Let $\gamma =h_{r}/h_{z}$ > 0 be the mesh ratio aspect.

Replacing the variables (x,y) by (r,z), and employing the approximation (6) to the PDE (19), we get

(20) $$\begin{array}{ccc} & & \left[\delta_{r}^{2}+\gamma^{2}\delta_{z}^{2}+\frac{1}{12}\left(1+\gamma^{2}\right)\delta_{r}^{2}\delta_{z}^{2}\right]u_{l,m}\\ & & -\;\frac{h_{r}^{2}}{12}[\left(1+\frac{h_{r}}{2r_{l}}\right)\frac{1}{r_{l+1}}{\hat{u}}_{r\;l+1,m}+\left(1-\frac{h_{r}}{2r_{l}}\right)\frac{1}{r_{l-1}}{\hat{u}}_{r\;l-1,m}\\ & & +\;\frac{1}{r_{l}}\left({\hat{u}}_{r\;l,m+1}+{\hat{u}}_{r\;l,m-1}+8{\hat{u}}_{r\;l,m}\right)+\frac{h_{r}}{2r_{l}}\left({\hat{u}}_{zz\;l+1,m}-{\hat{u}}_{zz\;l-1,m}\right)]\\ & & =\frac{h_{r}^{2}}{12}\left[\left(1+\frac{h_{r}}{2r_{l}}\right)g_{l+1,m}+\left(1-\frac{h_{r}}{2r_{l}}\right)g_{l-1,m}+g_{l,m+1}+g_{l,m-1}+8g_{l,m}\right] \end{array}$$

where $g_{l,m}=g\left(r_{l},z_{m}\right).$

The computational technique (20) is of $O(h_{r}^{4}+h_{r}^{2}h_{z}^{2}+h_{z}^{4})$. However, it is to be noticed that the coefficients $\frac{1}{r}$ and the function g(r,z) in (20) are not defined at (r,z)=(0,0). This means that the scheme (20) is unsuccessful for computation at l=1and m=1. The approximation usually declines in the neighborhood of the point (0,0) as $h_{r}\to 0$ and $h_{z}\to 0$. We conquer this circumstance by changing partially (20) by using the following approximations

(21) $$\frac{-1}{r_{l\pm 1}}=\frac{-1}{r_{l}}\pm \frac{h_{r}}{r_{l}^{2}}-\frac{h_{r}^{2}}{r_{l}^{3}}\pm O\left(h_{r}^{3}\right),$$

(22) $$g_{l\pm 1,m}=g_{l,m}\pm h_{r}g_{rl,m}+\frac{h_{r}^{2}}{2}g_{rrl,m}\pm O\left(h_{r}^{3}\right),$$

(23) $$g_{l,m\pm 1}=g_{l,m}\pm h_{z}g_{zl,m}+\frac{h_{z}^{2}}{2}g_{zzl,m}\pm O\left(h_{z}^{3}\right),$$

where $g_{l,m}=g\left(r_{l},z_{m}\right)$ etc.

Now, with the assistance of (21)–(23), from (20), we have

(24) $$\begin{array}{ccc} & & \left[1-\frac{h_{r}^{2}}{12r_{l}^{2}}\right]\delta_{r}^{2}u_{l,m}+\left[\frac{h_{r}}{2r_{l}}+\frac{h_{r}^{3}}{24r_{l}^{3}}\right]\left(2\mu_{r}\delta_{r}\right)u_{l,m}+\gamma^{2}\delta_{z}^{2}u_{l,m}+\frac{h_{r}}{24r_{l}}\left[1+\gamma^{2}\right]\left(\delta_{z}^{2}2\mu_{r}\delta_{r}\right)u_{l,m}\\ & & +\;\frac{1}{12}\left[1+\gamma^{2}\right]\delta_{r}^{2}\delta_{z}^{2}u_{l,m}=\frac{h_{r}^{2}}{12}\left[12g_{l,m}+h_{r}^{2}\left(g_{rrl,m}+\frac{1}{r_{l}}g_{rl,m}\right)+h_{z}^{2}g_{zzl,m}\right]. \end{array}$$

The modified scheme (24) is of $O(h_{r}^{4}+h_{r}^{2}h_{z}^{2}+h_{z}^{4})$. This modified scheme is suitable to solve EPDE (19) in *r-z* plane, where (0,0) is a boundary point. This has been illustrated through Example 4 in this article.

In a similar manner, we can derive modified method for nonlinear elliptic BVPs in *r-z* plane.

### Error analysis

In this subsection, the error analysis of the proposed discretization is discussed, when applied over the constant coefficient counterpart of the Eq. (1.1) viz.

(25) $$au_{xx}+bu_{yy}=\Phi \left(x,y,u,u_{x},u_{y}\right),$$

subject to the condition (1.2), where a,b are constants. We assume that $\gamma =\frac{h_{x}}{h_{y}}=$ constant, and hence show that the discretization (6) applied to (25) is $O(h_{x}^{4})$ convergent.

Applying (6) over the Eq. (25), we obtain

(26) $$\begin{array}{ccc} & & \left(\frac{a+\gamma^{2}b}{2}\right)U_{8}-\left(a-5\gamma^{2}b\right)U_{4}+\left(\frac{a+\gamma^{2}b}{2}\right)U_{6}-\left(\gamma^{2}b-5a\right)U_{2}-10\left(a+\gamma^{2}b\right)U_{0}\\ & & -\;\left(\gamma^{2}b-5a\right)U_{1}+\left(\frac{a+\gamma^{2}b}{2}\right)U_{7}-\left(a-5\gamma^{2}b\right)U_{3}+\left(\frac{a+\gamma^{2}b}{2}\right)U_{5}\\ & & =\frac{h^{2}}{2}\left[12{\hat{\hat{\Phi }}}_{l,m}+{\hat{\Phi }}_{l+1,m}+{\hat{\Phi }}_{l-1,m}+{\hat{\Phi }}_{l,m+1}+{\hat{\Phi }}_{l,m-1}-4{\tilde{\tilde{\Phi }}}_{l,m}\right]+{\hat{T}}_{l,m}, \end{array}$$

for each internal grid point $(x_{l},y_{m}$), with ${\hat{T}}_{l,m}=O\left(h_{x}^{6}\right)$.

Let for 1≤l,m≤N−1,

$M_{l,m}=\frac{h^{2}}{2}\left[12{\hat{\hat{\Phi }}}_{l,m}+{\hat{\Phi }}_{l+1,m}+{\hat{\Phi }}_{l-1,m}+{\hat{\Phi }}_{l,m+1}+{\hat{\Phi }}_{l,m-1}-4{\tilde{\tilde{\Phi }}}_{l,m}\right]$+boundary values, and $E_{l,m}=u_{l,m}-U_{l,m}$. Further, with *t* denoting the transpose and for $S=M,u,U,\;\bar{T}$ and E, we define

$$S={\left[S_{1,1},S_{2,1},\ldots ,S_{N-1,1},S_{1,2},S_{2,2},\ldots ,S_{N-1,2},\ldots ,S_{1,N-1},S_{2,N-1},\ldots ,S_{N-1,N-1}\right]}_{{\left(N-1\right)}^{2}\times \;1}^{t}.$$

Then, as (l,m) are varied such that 1≤l,m≤N−1, let us re-write Eq. (26) as

(27) Du+M(u)=0,

for

$K={\left[\gamma^{2}b-5a\;10\left(a+\gamma^{2}b\right)\;\gamma^{2}b-5a\right]}_{\left(N-1)\;\times \;(N-1\right)}$ (Tri-diagonal matrix),

$B={\left[-\left(\frac{a+\gamma^{2}b}{2}\right)\;a-5\gamma^{2}b\;-\left(\frac{a+\gamma^{2}b}{2}\right)\;\right]}_{\left(N-1)\;\times \;(N-1\right)}\;$(Tri-diagonal matrix),

$D={\left[B\;K\;B\right]}_{{\left(N-1\right)}^{2}\times {\left(N-1\right)}^{2}}$ (Block Tri-diagonal matrix).

Here, we have made the following assumptions

(28) $$\gamma^{2}b-5a>0,$$

(29) $$a-5\gamma^{2}b>0.$$

It is easy to see that the above two conditions yield a>0 and b>0. This renders strictly positive diagonal entries and strictly negative off-diagonal entries for the matrix D.

Since $U_{l,m}$ is assumed to be the analytical solution calculated at each grid point $\left(x_{l},y_{m}\right)$,

(30) $$\mathrm{DU}+M\left(U\right)+\bar{T}=0.$$

It is to be noted that for every pair of (l,m) such that 1≤l,m≤N, ${\bar{T}}_{l,m}=O\left(h_{x}^{6}\right)$.

Now, let us define

$${\hat{\phi }}_{l\pm 1,m}=\phi \left(x_{l\pm 1},y_{m},u_{l\pm 1,m},{\hat{u}}_{xl\pm 1,m},{\hat{u}}_{yl\pm 1,m}\right)≅{\hat{\Phi }}_{l\pm 1,m},$$

$${\hat{\phi }}_{l,m\pm 1}=\phi \left(x_{l},y_{m\pm 1},u_{l,m\pm 1},{\hat{u}}_{xl,m\pm 1},{\hat{u}}_{yl,m\pm 1}\right)≅{\hat{\Phi }}_{l,m\pm 1},$$

$${\hat{\hat{\phi }}}_{l,m}=\phi \left(x_{l},y_{m},u_{l,m},{\hat{\hat{u}}}_{xl,m},{\hat{\hat{u}}}_{yl,m}\right)≅{\hat{\hat{\Phi }}}_{l,m},$$

$${\tilde{\tilde{\phi }}}_{l,m}=\phi \left(x_{l},y_{m},u_{l,m},{\tilde{\tilde{u}}}_{xl,m},{\tilde{\tilde{u}}}_{yl,m}\right)≅{\tilde{\tilde{\Phi }}}_{l,m}.$$

Then, we can easily obtain the following

(31.1) $${\hat{\phi }}_{l\pm 1,m}-{\hat{\Phi }}_{l\pm 1,m}=\left(u_{l\pm 1,m}-U_{l\pm 1,m}\right)\rho_{l\pm 1,m}^{\left(1\right)}+\left({\hat{u}}_{xl\pm 1,m}-{\hat{U}}_{xl\pm 1,m}\right)\alpha_{l\pm 1,m}^{\left(1\right)}+\left({\hat{u}}_{yl\pm 1,m}-{\hat{U}}_{yl\pm 1,m}\right)\beta_{l\pm 1,m}^{\left(1\right)},$$

(31.2) $${\hat{\phi }}_{l,m\pm 1}-{\hat{\Phi }}_{l,m\pm 1}=\left(u_{l,m\pm 1}-U_{l,m\pm 1}\right)\rho_{l,m\pm 1}^{\left(2\right)}+\left({\hat{u}}_{xl,m\pm 1}-{\hat{U}}_{xl,m\pm 1}\right)\alpha_{l,m\pm 1}^{\left(2\right)}+\left({\hat{u}}_{yl,m\pm 1}-{\hat{U}}_{yl,m\pm 1}\right)\beta_{l,m\pm 1}^{\left(2\right)},$$

(31.3) $${\hat{\hat{\phi }}}_{l,m}-{\hat{\hat{\Phi }}}_{l,m}=\left(u_{l,m}-U_{l,m}\right)\rho_{l,m}^{\left(3\right)}+\left({\hat{\hat{u}}}_{xl,m}-{\hat{\hat{U}}}_{xl,m}\right)\alpha_{l,m}^{\left(3\right)}+\left({\hat{\hat{u}}}_{yl,m}-{\hat{\hat{U}}}_{yl,m}\right)\beta_{l,m}^{\left(3\right)},$$

(31.4) $${\tilde{\tilde{\phi }}}_{l,m}-{\tilde{\tilde{\Phi }}}_{l,m}=\left(u_{l,m}-U_{l,m}\right)\rho_{l,m}^{\left(3\right)}+\left({\tilde{\tilde{u}}}_{xl,m}-{\tilde{\tilde{U}}}_{xl,m}\right)\alpha_{l,m}^{\left(3\right)}+\left({\tilde{\tilde{u}}}_{yl,m}-{\tilde{\tilde{U}}}_{yl,m}\right)\beta_{l,m}^{\left(3\right)},$$

for suitable $Q_{l\pm 1,m}^{\left(1\right)}$, $Q_{l,m\pm 1}^{\left(2\right)}$ and $Q_{l,m}^{\left(3\right)}$, where Q=ρ, α and β.Now, taking Q=α and β, we obtain

(32.1) $$Q_{l\pm 1,m}^{\left(1\right)}=Q_{l,m}^{\left(1\right)}\pm h_{x}{\left(\frac{\partial Q^{\left(1\right)}}{\partial x}\right)}_{l,m}+O\left(h_{x}^{2}\right),$$

(32.2) $$Q_{l,m\pm 1}^{\left(2\right)}=Q_{l,m}^{\left(2\right)}\pm \frac{h_{x}}{\gamma }{\left(\frac{\partial Q^{\left(2\right)}}{\partial y}\right)}_{l,m}+O\left(h_{x}^{2}\right),$$

and

(33.1) $$\rho_{l\pm 1,m}^{\left(1\right)}=\rho_{l,m}^{\left(1\right)}\pm O\left(h_{x}\right),$$

(33.2) $$\rho_{l,m\pm 1}^{\left(2\right)}=\rho_{l,m}^{\left(2\right)}\pm O\left(h_{x}\right).$$

Thus, Eqs. (31.1)–(33.2) fetch us the following

(34) M(u)−M(U)=PE,

for $P=(P_{r,s})$, [$1\leq r\leq {\left(N-1\right)}^{2},\;1\leq s\leq {\left(N-1\right)}^{2}]$ being the block tri-diagonal matrix with the following entries

$$P_{\left(m-1\right)\left(N-1\right)+l,\;\left(m-1\right)\left(N-1\right)+l}=h_{x}^{2}[4\rho_{l,m}^{\left(3\right)}+\frac{1}{a}\alpha_{l,m}^{\left(1\right)}\alpha_{l,m}^{\left(3\right)}+\frac{1}{b}\beta_{l,m}^{\left(2\right)}\beta_{l,m}^{\left(3\right)}-2{\left(\frac{\partial \alpha^{\left(1\right)}}{\partial x}\right)}_{l,m}$$

$$-2{\left(\frac{\partial \beta^{\left(2\right)}}{\partial y}\right)}_{l,m}]+O\left(h_{x}^{4}\right),\left[1\leq l\leq N-1,\;1\leq m\leq N-1\right],$$

$$P_{\left(m-1\right)\left(N-1\right)+l,\;\left(m-1\right)\left(N-1\right)+l\pm 1}=\frac{h_{x}}{2}\left[\pm \alpha_{l,m}^{\left(1\right)}\pm \left(4-\frac{b\gamma^{2}}{a}\right)\alpha_{l,m}^{\left(3\right)}\right]$$

$$+\frac{h_{x}^{2}}{2}\left[2{\left(\frac{\partial \alpha^{\left(1\right)}}{\partial x}\right)}_{l,m}+\rho_{l,m}^{\left(1\right)}-\frac{1}{a}\alpha_{l,m}^{\left(1\right)}\alpha_{l,m}^{\left(3\right)}\right]+O\left(h_{x}^{3}\right),$$

[1≤l≤N−2,1≤m≤N−1],[2≤l≤N−1,1≤m≤N−1],

$$P_{\left(m-1\right)\left(N-1\right)+l,\left(m-1\pm 1\right)\left(N-1\right)+l}=\frac{h_{x}}{2}\left[\pm \gamma \beta_{l,m}^{\left(2\right)}\pm \left(4\gamma -\frac{a}{b\gamma }\right)\beta_{l,m}^{\left(3\right)}\right]$$

$$+\frac{h_{x}^{2}}{2}\left[2{\left(\frac{\partial \beta^{\left(2\right)}}{\partial y}\right)}_{l,m}+\rho_{l,m}^{\left(2\right)}-\frac{1}{b}\beta_{l,m}^{\left(2\right)}\beta_{l,m}^{\left(3\right)}\right]+O\left(h_{x}^{3}\right),$$

[1≤l≤N−1,1≤m≤N−2],[1≤l≤N−1,2≤m≤N−1],

$$P_{\left(m-1\right)\left(N-1\right)+l,m\left(N-1\right)+l\pm 1}=\frac{h_{x}}{4}\left(\gamma \beta_{l,m}^{\left(1\right)}\pm \alpha_{l,m}^{\left(2\right)}+\frac{a}{b\gamma }\beta_{l,m}^{\left(3\right)}\pm \frac{b\gamma^{2}}{a}\alpha_{l,m}^{\left(3\right)}\right)$$

$$+\frac{h_{x}^{2}}{8}\left[\pm 2\gamma {\left(\frac{\partial \beta^{\left(1\right)}}{\partial x}\right)}_{l,m}\pm \frac{2}{\gamma }{\left(\frac{\partial \alpha^{\left(2\right)}}{\partial y}\right)}_{l,m}\mp \frac{\gamma }{a}\beta_{l,m}^{\left(1\right)}\alpha_{l,m}^{\left(3\right)}\mp \frac{1}{b\gamma }\alpha_{l,m}^{\left(2\right)}\beta_{l,m}^{\left(3\right)}\right]+O\left(h_{x}^{3}\right),$$

[1≤l≤N−2,1≤m≤N−2],[2≤l≤N−1,1≤m≤N−2],

$$P_{\left(m-1\right)\left(N-1\right)+l,\left(m-2\right)\left(N-1\right)+l\pm 1}=\frac{h_{x}}{4}\left(-\gamma \beta_{l,m}^{\left(1\right)}\pm \alpha_{l,m}^{\left(2\right)}-\frac{a}{b\gamma }\beta_{l,m}^{\left(3\right)}\pm \frac{b\gamma^{2}}{a}\alpha_{l,m}^{\left(3\right)}\right)$$

$$+\frac{h_{x}^{2}}{8}\left[\mp 2\gamma {\left(\frac{\partial \beta^{\left(1\right)}}{\partial x}\right)}_{l,m}\pm \frac{2}{\gamma }{\left(\frac{\partial \alpha^{\left(2\right)}}{\partial y}\right)}_{l,m}\pm \frac{\gamma }{a}\beta_{l,m}^{\left(1\right)}\alpha_{l,m}^{\left(3\right)}\pm \frac{1}{b\gamma }\alpha_{l,m}^{\left(2\right)}\beta_{l,m}^{\left(3\right)}\right]+O\left(h_{x}^{3}\right),$$

[1≤l≤N−2,2≤m≤N−1],[2≤l≤N−1,2≤m≤N−1].

Ignoring the existence of the round-off errors, relations (27), (30) and (34) give us the following equation

(35) $$\left(D+P\right)E=\bar{T},$$

called the error equation.

Let $\bar{\Omega }=\Omega_{h}\cup \Gamma$, and

$\rho_{*}=$Min $\left(\frac{\partial f}{\partial u}\right)$ on $\bar{\Omega }$ and $\rho^{*}=$Max $\left(\frac{\partial f}{\partial u}\right)$ on $\bar{\Omega }$then

$$0<\rho_{*}\leq \rho_{l\pm 1,m}^{\left(1\right)},\;\rho_{l,m\pm 1}^{\left(2\right)},\rho_{l,m}^{\left(3\right)}\leq \rho^{*}.$$

Also, for Q=α and β, let

$$0<\left|Q_{l\pm 1,m}^{\left(1\right)}\right|,\left|Q_{l,m\pm 1}^{\left(2\right)}\right|,\left|Q_{l,m}^{\left(3\right)}\right|\leq Q$$

and

$|{\left(\frac{\partial Q^{\left(1\right)}}{\partial x}\right)}_{l,m}|\leq Q^{\left(1\right)}$ and $|{\left(\frac{\partial Q^{\left(2\right)}}{\partial y}\right)}_{l,m}|\leq Q^{\left(2\right)}$where Q, $Q^{\left(1\right)}$ and $Q^{\left(2\right)}$ are some positive constants.

For sufficiently small $h_{x}$, we obtain

$$\left|P_{\left(m-1\right)\left(N-1\right)+l,\;\left(m-1\right)\left(N-1\right)+l}\right|<10a+\gamma^{2}b,\;\left[1\leq l\leq N-1,\;1\leq m\leq N-1\right],$$

$$\left|P_{\left(m-1\right)\left(N-1\right)+l,\;\left(m-1\right)\left(N-1\right)+l\pm 1}\right|<\gamma^{2}b-5a,$$

[1≤l≤N−2,1≤m≤N−1],[2≤l≤N−1,1≤m≤N−1],

$$\left|P_{\left(m-1\right)\left(N-1\right)+l,\left(m-1\pm 1\right)\left(N-1\right)+l}\right|<\gamma^{2}b-5a,$$

[1≤l≤N−1,1≤m≤N−2],[1≤l≤N−1,2≤m≤N−1],

$$\left|P_{\left(m-1\right)\left(N-1\right)+l,m\left(N-1\right)+l\pm 1}\right|<-\left(\frac{a+\gamma^{2}b}{2}\right),$$

[1≤l≤N−2,1≤m≤N−2],[2≤l≤N−1,1≤m≤N−2],

$$\left|P_{\left(m-1\right)\left(N-1\right)+l,\left(m-2\right)\left(N-1\right)+l\pm 1}\right|<-\left(\frac{a+\gamma^{2}b}{2}\right),$$

[1≤l≤N−2,2≤m≤N−1],[2≤l≤N−1,2≤m≤N−1].

Further, it is easy to see that the directed graph of the matrix D+P is strongly connected. Therefore, by Varga [43], we conclude that D+P is an irreducible matrix.

Let the sum of elements in the $k^{th}$ row of the matrix D+P be denoted by $S_{k}.$ Then, for k=1,N−1, we obtain the following

(36) $$S_{k}=\frac{11\left(\gamma^{2}b+a\right)}{2}+\frac{h_{x}^{2}}{2}\left(8\rho_{k,1}^{\left(3\right)}+\rho_{k,1}^{\left(1\right)}+\rho_{k,1}^{\left(2\right)}\right)+\frac{h_{x}}{8}\left(b_{k}+h_{x}c_{k}\right)+O\left(h_{x}^{3}\right),$$

where

$$b_{k}=\pm 4\alpha_{k,1}^{\left(1\right)}\pm 2\alpha_{k,1}^{\left(2\right)}\pm \left(16-\frac{2b\gamma^{2}}{a}\right)\alpha_{k,1}^{\left(3\right)}+2\gamma \beta_{k,1}^{\left(1\right)}+4\gamma \beta_{k,1}^{\left(2\right)}+\left(16\gamma -\frac{2a}{b\gamma }\right)\beta_{k,1}^{\left(3\right)},$$

$$\begin{array}{ccc} c_{k} & = & \frac{4}{a}\alpha_{k,1}^{\left(1\right)}\alpha_{k,1}^{\left(3\right)}+\frac{4}{b}\beta_{k,1}^{\left(2\right)}\beta_{k,1}^{\left(3\right)}\mp \frac{\gamma }{a}\beta_{k,1}^{\left(1\right)}\alpha_{k,1}^{\left(3\right)}\mp \frac{1}{b\gamma }\beta_{k,1}^{\left(3\right)}\alpha_{k,1}^{\left(2\right)}-8{\left(\frac{\partial \alpha^{\left(1\right)}}{\partial x}\right)}_{k,1}\\ & & -\;8{\left(\frac{\partial \beta^{\left(2\right)}}{\partial y}\right)}_{k,1}\;\pm 2\gamma {\left(\frac{\partial \beta^{\left(1\right)}}{\partial x}\right)}_{k,1}\pm \frac{2}{\gamma }{\left(\frac{\partial \alpha^{\left(2\right)}}{\partial y}\right)}_{k,1}. \end{array}$$

(37) $$S_{\left(N-1\right)\left(N-2\right)+k}=\frac{11\left(\gamma^{2}b+a\right)}{2}+\frac{h_{x}^{2}}{2}\left(8\rho_{k,N-1}^{\left(3\right)}+\rho_{k,N-1}^{\left(1\right)}+\rho_{k,N-1}^{\left(2\right)}\right)+\frac{h_{x}}{8}\left(b_{k}+h_{x}c_{k}\right)+O\left(h_{x}^{3}\right),$$

where

$$b_{k}=\pm 4\alpha_{k,N-1}^{\left(1\right)}\pm 2\alpha_{k,N-1}^{\left(2\right)}\pm \left(16-\frac{2b\gamma^{2}}{a}\right)\alpha_{k,N-1}^{\left(3\right)}+2\gamma \beta_{k,N-1}^{\left(1\right)}+4\gamma \beta_{k,N-1}^{\left(2\right)}$$

$$+\left(16\gamma -\frac{2a}{b\gamma }\right)\beta_{k,N-1}^{\left(3\right)},$$

$$c_{k}=-\frac{4}{a}\alpha_{k,N-1}^{\left(1\right)}\alpha_{k,N-1}^{\left(3\right)}-\frac{4}{b}\beta_{k,N-1}^{\left(2\right)}\beta_{k,N-1}^{\left(3\right)}\pm \frac{\gamma }{a}\beta_{k,N-1}^{\left(1\right)}\alpha_{k,N-1}^{\left(3\right)}\pm \frac{1}{b\gamma }\beta_{k,N-1}^{\left(3\right)}\alpha_{k,N-1}^{\left(2\right)}$$

$$+8{\left(\frac{\partial \alpha^{\left(1\right)}}{\partial x}\right)}_{k,N-1}+8{\left(\frac{\partial \beta^{\left(2\right)}}{\partial y}\right)}_{k,N-1}\mp 2\gamma {\left(\frac{\partial \beta^{\left(1\right)}}{\partial x}\right)}_{k,N-1}\mp \frac{2}{\gamma }{\left(\frac{\partial \alpha^{\left(2\right)}}{\partial y}\right)}_{k,N-1}.$$

For 2≤q≤N−2

(38) $$S_{\left(q-1\right)\left(N-1\right)+k}=6a+\frac{h_{x}^{2}}{2}\left(8\rho_{k,q}^{\left(3\right)}+\rho_{k,q}^{\left(1\right)}+2\rho_{k,q}^{\left(2\right)}\right)+\frac{h_{x}}{2}\left[b_{\left(q-1\right)\left(N-1\right)+k}+h_{x}c_{\left(q-1\right)\left(N-1\right)+k}\right]+O\left(h_{x}^{3}\right),$$

where

$$b_{\left(q-1\right)\left(N-1\right)+k}=\pm \alpha_{k,q}^{\left(2\right)}\pm \alpha_{k,q}^{\left(1\right)}\pm 4\alpha_{k,q}^{\left(3\right)},$$

$$c_{\left(q-1\right)\left(N-1\right)+k}=-2{\left(\frac{\partial \alpha^{\left(1\right)}}{\partial x}\right)}_{k,q}+\frac{1}{a}\alpha_{k,q}^{\left(1\right)}\alpha_{k,q}^{\left(3\right)}.$$

For 2≤r≤N−2

(39) $$S_{\left(k-1\right)\left(N-1\right)+r}=6\gamma^{2}b+\frac{h_{x}^{2}}{2}\left(8\rho_{r,k}^{\left(3\right)}+2\rho_{r,k}^{\left(1\right)}+\rho_{r,k}^{\left(2\right)}\right)+\frac{h_{x}}{2}\left[b_{\left(k-1\right)\left(N-1\right)+r}+h_{x}c_{\left(k-1\right)\left(N-1\right)+r}\right]+O\left(h_{x}^{3}\right),$$

where

$$b_{\left(k-1\right)\left(N-1\right)+r}=\pm \gamma \beta_{r,k}^{\left(1\right)}\pm \gamma \beta_{r,k}^{\left(2\right)}\pm 4\gamma \beta_{r,k}^{\left(3\right)},$$

$$c_{\left(k-1\right)\left(N-1\right)+r}=-2{\left(\frac{\partial \beta^{\left(2\right)}}{\partial y}\right)}_{r,k}+\frac{1}{b}\beta_{r,k}^{\left(2\right)}\beta_{r,k}^{\left(3\right)}.$$

Finally, for 2≤q≤N−2 and 2≤r≤N−2

(40) $$S_{\left(r-1\right)\left(N-1\right)+q}=h_{x}^{2}\left(4\rho_{q,r}^{\left(3\right)}+\rho_{q,r}^{\left(1\right)}+\rho_{q,r}^{\left(2\right)}\right)+O\left(h_{x}^{4}\right).$$

From the Eqs. (36)–(40), we get

$$\left|b_{k}\right|\leq \left(22+\frac{2b\gamma^{2}}{a}\right)\alpha +\left(22\gamma +\frac{2a}{b\gamma }\right)\beta ,$$

$$\left|c_{k}\right|\leq 4\frac{\alpha^{2}}{a}+4\frac{\beta^{2}}{b}+\left(\frac{\gamma }{a}+\frac{1}{b\gamma }\right)\alpha \beta +8\left[\alpha^{\left(1\right)}+\beta^{\left(2\right)}\right]+2\left[\gamma \beta^{\left(1\right)}+\frac{1}{\gamma }\alpha^{\left(2\right)}\right],$$

for $k=1,N-1,\left(N-1\right)\left(N-2\right)+1,{\left(N-1\right)}^{2}.$

$$\left|b_{k}\right|\leq 6\alpha ,$$

$$\left|c_{k}\right|\leq 2\alpha^{\left(1\right)}+\frac{\alpha^{2}}{a},$$

for k=(q−1)(N−1)+1 and q(N−1), where 2≤q≤N−2.

$$\left|b_{k}\right|\leq 6\gamma \beta ,$$

$$\left|c_{k}\right|\leq 2\beta^{\left(2\right)}+\frac{\beta^{2}}{b},$$

for k=r and (N−2)(N−1)+r, where 2≤r≤N−2.

For sufficiently small $h_{x}$, we obtain

$S_{k}>5h_{x}^{2}\rho_{*}$ [for $k=1,N-1,\left(N-1\right)\left(N-2\right)+1,{\left(N-1\right)}^{2}]$,

$S_{k}>\frac{11}{2}h_{x}^{2}\rho_{*}$ [for k=(q−1)(N−1)+1 and q(N−1), where 2≤q≤N−2],

$S_{k}>\frac{11}{2}h_{x}^{2}\rho_{*}$ [for k=r and (N−2)(N−1)+r, where 2≤r≤N−2],

$S_{(r-1)(N-2)+q}\geq 6h_{x}^{2}\rho_{*}$ [for 2≤r≤N−2 and 2≤q≤N−2].

Thus, D+P is monotone for sufficiently small value of $h_{x}$. Hence, its inverse exists (Varga [43]). Let ${\left(D+P\right)}^{-1}=J^{-1}$, where for $1\leq r\leq {\left(N-1\right)}^{2}$ and $1\leq s\leq {\left(N-1\right)}^{2}$, $J=(J_{r,s})$.

Since

$\sum_{r=1}^{{\left(N-1\right)}^{2}}J_{p,r}S_{r}=1,\text{for}1\leq p\leq {\left(N-1\right)}^{2}$, we obtain

(41) $$J_{p,k}\leq \frac{1}{S_{k}}<\frac{1}{5h_{x}^{2}\rho_{*}}\left[\text{for}k=1,\;N-1,\;\left(N-1\right)\left(N-2\right)+1,\;{\left(N-1\right)}^{2}\right],$$

(42) $$\sum_{q=2}^{N-2}J_{p,k}<\frac{2}{11h_{x}^{2}\rho_{*}}\left[\text{for}k=\left(q-1\right)\left(\;N-1\right)+1,q\left(N-1\right),2\leq q\leq N-2\right],$$

(43) $$\sum_{r=2}^{N-2}J_{p,k}<\frac{2}{11h_{x}^{2}\rho_{*}}\left[\text{for}k=r,\;\left(N-1\right)\left(\;N-2\right)+r,2\leq r\leq N-2\right],$$

(44) $$\sum_{q=2}^{N-2}\sum_{r=2}^{N-2}J_{p,k}\leq \frac{1}{6h_{x}^{2}\rho_{*}}\left[fork=\left(r-1\right)\left(\;N-1\right)+q,2\leq q\leq N-2,2\leq r\leq N-2\right].$$

Now, we re-write Eq. (35) as

(45) ∥E∥≤∥J∥∥T∥,

where

(46) $$\begin{array}{ccc} J & = & Max_{1\leq p\leq {\left(N-1\right)}^{2}}[\left(J_{p,1}+\sum_{q=2}^{N-2}J_{p,q}+J_{p,N-1}\right)+(\sum_{q=2}^{N-2}J_{p,\left(q-1\right)\left(N-1\right)+1}+\sum_{q=2}^{N-2}\sum_{r=2}^{N-2}J_{p,\left(q-1\right)\left(N-1\right)+r}+\sum_{q=2}^{N-2}J_{p,q\left(N-1\right)})\\ & & +\left(J_{p,\left(N-1\right)\left(N-2\right)+1}+\sum_{q=2}^{N-2}J_{p,\left(N-1\right)\left(N-2\right)+q}+J_{p,{\left(N-1\right)}^{2}}\right)] \end{array}$$

Using Eqs. (41)–(44) and (46) in Eq. (45), for appropriately small value of $h_{x}$, we get

(47) $$∥E∥\leq O\left(h_{x}^{4}\right).$$

Thus, the proposed technique applied over Eq. (25) is $O(h_{x}^{4})$ convergent.

## Method validation

Five standard problems of physical significance are numerically solved in this section. The closed-form solutions of these problems are available. The functions on the right-hand side and the DBCs can be attained from the analytical solution as a test procedure. Both nonlinear and linear difference equations in block forms are solved with the aid of suitable iterative methods ([38,43,44,46]). During computation, the iterations are terminated when $|u^{\left(k+1\right)}-u^{\left(k\right)}|\leq 10^{-12}$ is achieved. The initial solution is chosen as vector **0** for all iterative methods and the Maximum Absolute Errors (MAEs) are computed. All the results were computed using MATLAB codes.

Example 1 (Poisson equation in r−z plane)

(48) $$u_{rr}+u_{zz}+\frac{1}{r}u_{r}=f\left(r,z\right),0<\;z<e,0<r<1.$$

**Fig. 2:**
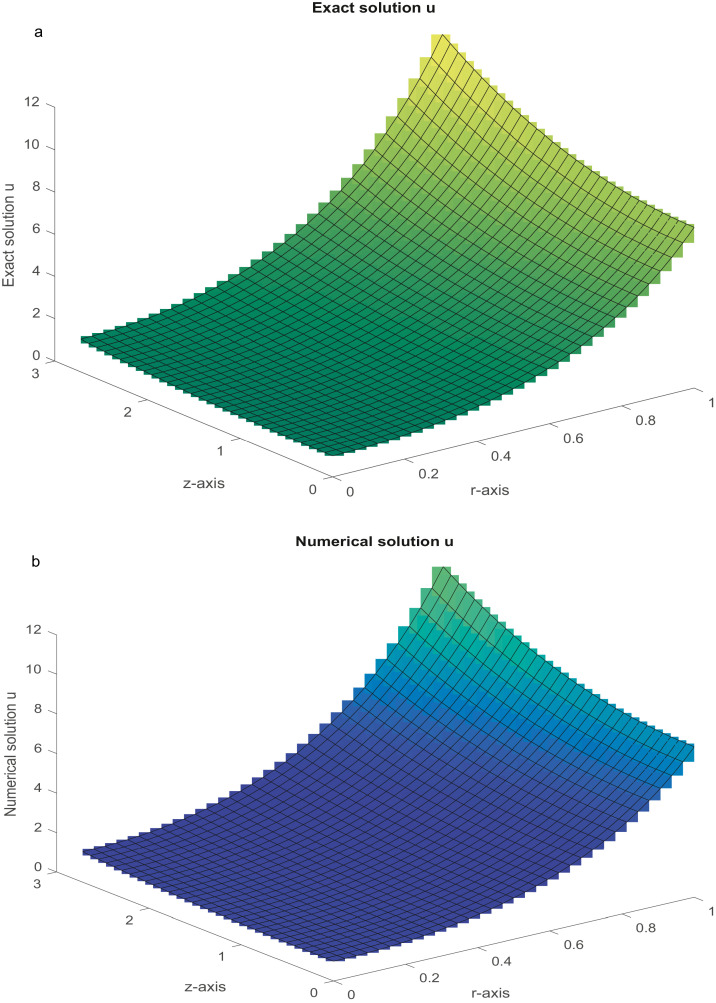
a: The graph of analytical solution with *N_x_* *=* *N_y_* = 31. Fig. 2b: The graph of numerical solution with $N_{x}=N_{y}=31$.

The analytical solution isu(r,z)=coshr.coshz. By the help of the proposed method (6) and using the technique discussed in [24], we solve the Eq. (48). The MAEs are presented in Table 1. Figs. 2a and b depict the plots of the analytical and numerical solutions, respectively, for $N_{x}=N_{y}$=31.

**Table 1:** (Example 1): The MAEs.

$N_{x}=N_{y}$	Proposed FDM	Method [49]	Method [56]
15	1.2098(−08)	2.1503(−08)	1.0607(−03)
CPU time (sec)	(0.1518)		
31	7.7217(−10)	1.5357(−09)	2.6858(−04)
CPU time (sec)	(0.8118)		
63	4.8001(−11)	9.6354(−11)	6.8253(−05)
CPU time (sec)	(6.4516)

Example 2 (Burgers' equation)

(49) $$\varepsilon \left(u_{xx}+u_{yy}\right)=u\left(u_{x}+u_{y}\right)+g\left(x,y\right),0<x<\frac{1}{\sqrt{2}},\;0<y<1.$$

**Fig. 3:**
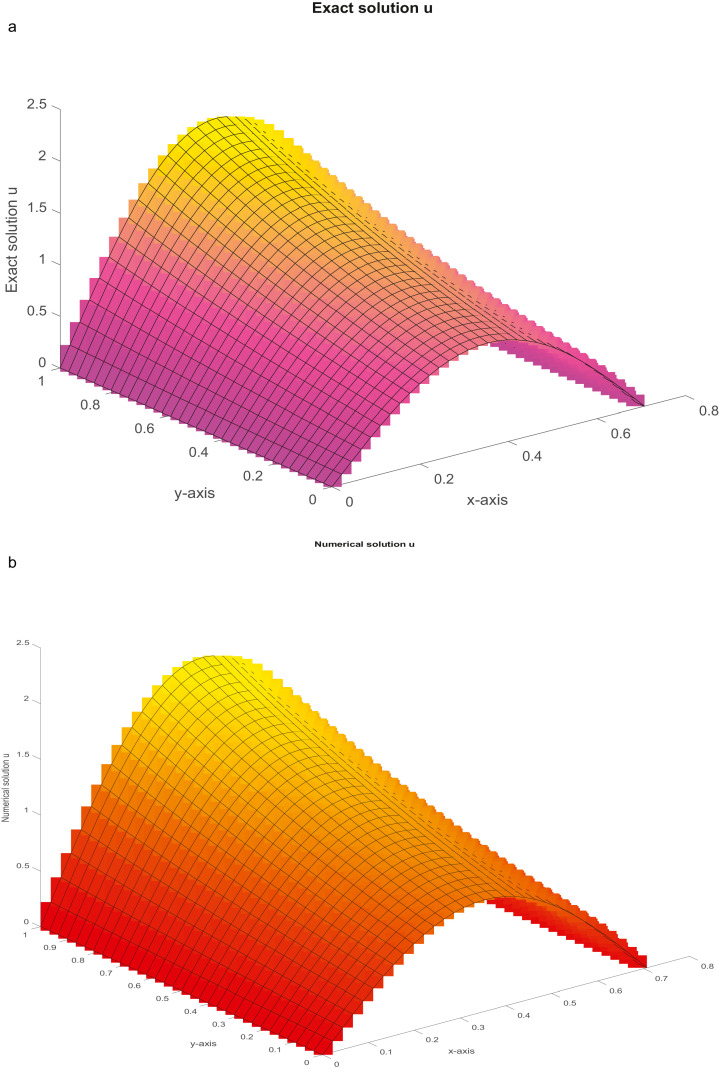
a: The graph of closed-form solution with ε=0.01 and *N_x_* = *N_y_* = 31. Fig. 3b: The graph of numerical solution with ε=0.01 and *N_x_* = *N_y_* = 31.

For Eq. (49), the closed-form solution is $u=e^{x}.\;\text{sin}\left(\pi y\right)$. The MAEs for the solution u are presented in Table 2. The plots of the closed-form and numerical solutions are depicted in Figs. 3a and b, respectively, for ε=0.01 and $N_{x}=N_{y}$=31.

**Table 2:** (Example 2): The MAEs.

$N_{x}=N_{y}$	Proposed FDM			Method [49]			Method [56]	
	ε=0.1	ε=0.01	ε=0.001	ε=0.1	ε=0.01	ε=0.001	ε=0.1	ε=0.01,0.001
15 CPU time (sec)	2.3721(−07) (0.0795)	5.3311(−06) (1.1876)	2.2039(−05) (1.2340)	7.6613(−07)	1.2494(−05)	3.9373(−05)	1.6176(−03)	Unstable
31 CPU time (sec)	1.4791(−08) (0.3136)	3.3019(−07) (2.2133)	1.3712(−06) (2.3011)	4.2417(−08)	7.7677(−07)	2.2758(−06)	3.8695(−04)	Unstable
63 CPU time (sec)	9.2983(−10) (2.5563)	2.0867(−08) (8.2431)	8.6777(−08) (9.1661)	2.6162(−09)	4.7816(−08)	1.4042(−07)	9.4937(−05)	Unstable

Example 3 (NS equations in cartesian coordinates)

(50.1) $$\frac{1}{R_{e}}\left(u_{xx}+u_{yy}\right)=uu_{x}+vu_{y}+f\left(x,y\right),0<x<1,\;0<y<\frac{1}{\sqrt{2}},$$

(50.2) $$\frac{1}{R_{e}}\left(v_{xx}+v_{yy}\right)=uv_{x}+vv_{y}+g\left(x,y\right),0<x<1,\;0<y<\frac{1}{\sqrt{2}}.$$

**Fig. 4:**
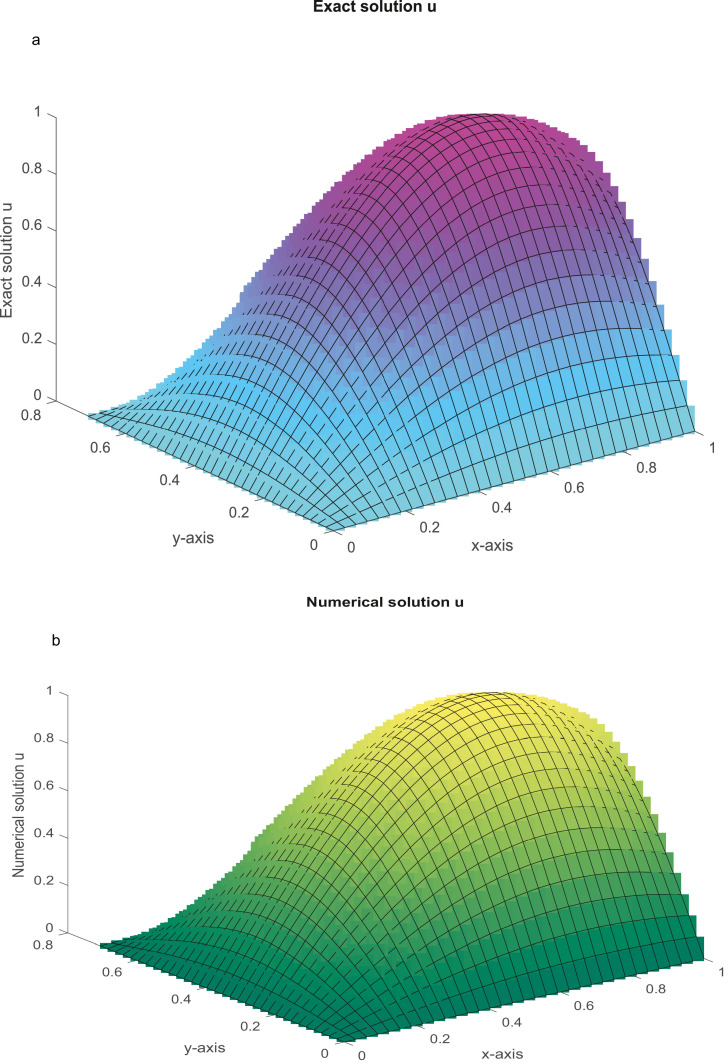

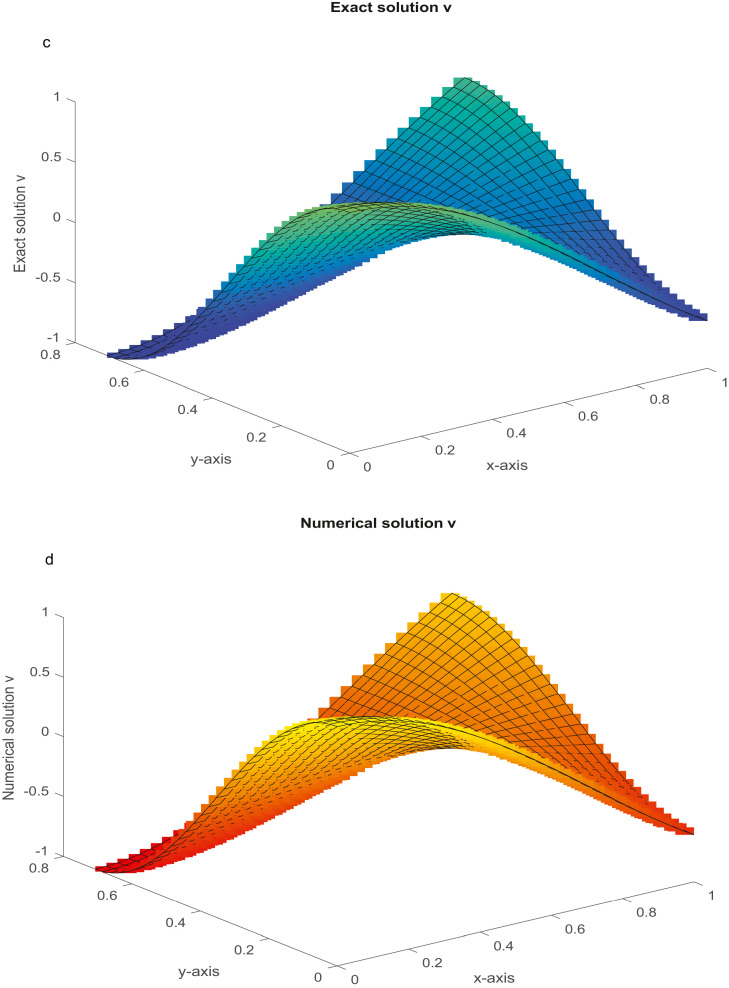
a: The graph of analytical solution, u, with *R_e_*=10^4^ and *N_x_* = *N_y_* = 31. Fig. 4b: The graph of numerical solution, u, with *R_e_* = 10^4^ and *N_x_* = *N_y_* = 31. Fig. 4c: The graph of analytical solution, *v,* with *R_e_* = 10^4^ and $N_{x}$ = *N_y_* = 31. Fig. 4d: The graph of numerical solution, *v*, with *R_e_* = 10^4^ and *N_x_* = *N_y_* = 31.

The analytical solutions are u=sin(πx)sin(πy),v=cos(πx)cos(πy). For varying values of *R_e_*, the MAEs are reported in Table 3. The analytical and numerical solutions are plotted in Figs. 4a-d for $N_{x}=N_{y}$=31 and $R_{e}=10^{4}.$

Example 4 (NS equations in cylindrical polar coordinates)

(51.1) $$\frac{1}{R_{e}}\left(u_{rr}+\frac{1}{r}u_{r}+u_{zz}-\frac{1}{r^{2}}u\right)=uu_{r}+vu_{z}+f\left(r,z\right),0<r<1,0<z<\sqrt{2},$$

(51.2) $$\frac{1}{R_{e}}\left(v_{rr}+\frac{1}{r}v_{r}+v_{zz}\right)=uv_{r}+vv_{z}+g\left(r,z\right),0<r<1,0<z<\sqrt{2}.$$

**Table 3:** (Example 3): The MAEs.

$N_{x}=N_{y}$	Proposed FDM			Method [49]			Method [56]	
	$R_{e}=10$	$R_{e}=10^{2}$	$R_{e}=10^{4}$	$R_{e}=10$	$R_{e}=10^{2}$	$R_{e}=10^{4}$	$R_{e}=10$	$R_{e}=10^{2},10^{4}$
15 *u*	2.3759(−06)	2.2705(−05)	1.6686(−03)	3.0563(−06)	7.1474(−05)	4.6314(−03)	1.2141(−03)	Unstable
*v*	6.5170(−07)	7.1474(−06)	1.4175(−03)	2.2897(−06)	2.3107(−05)	3.0187(−03)	6.5296(−04)	
CPU time (sec)	(2.2386)	(2.8835)	(3.2231)					
31 *u*	1.5405(−07)	1.3628(−06)	1.1188(−04)	1.9065(−07)	4.3910(−06)	2.8929(−04)	2.9334(−04)	Unstable
*v*	4.0903(−08)	4.3910(−07)	8.9654(−05)	1.4276(−07)	1.4416(−06)	1.8831(−04)	1.5696(−04)	
CPU time (sec)	(6.1298)	(7.4762)	(8.2342)					
63 *u*	9.7192(−09)	8.5442(−08)	7.0458(−06)	1.1845(−08)	1.7156(−07)	1.8045(−05)	7.2146(−05)	Unstable
*v*	2.5694(−09)	2.7756(−08)	5.6432(−06)	8.9755(−09)	9.0650(−08)	1.1785(−05)	3.8553(−05)	
CPU time (sec)	(25.3462)	(28.1260)	(31.7743)

**Fig. 5:**
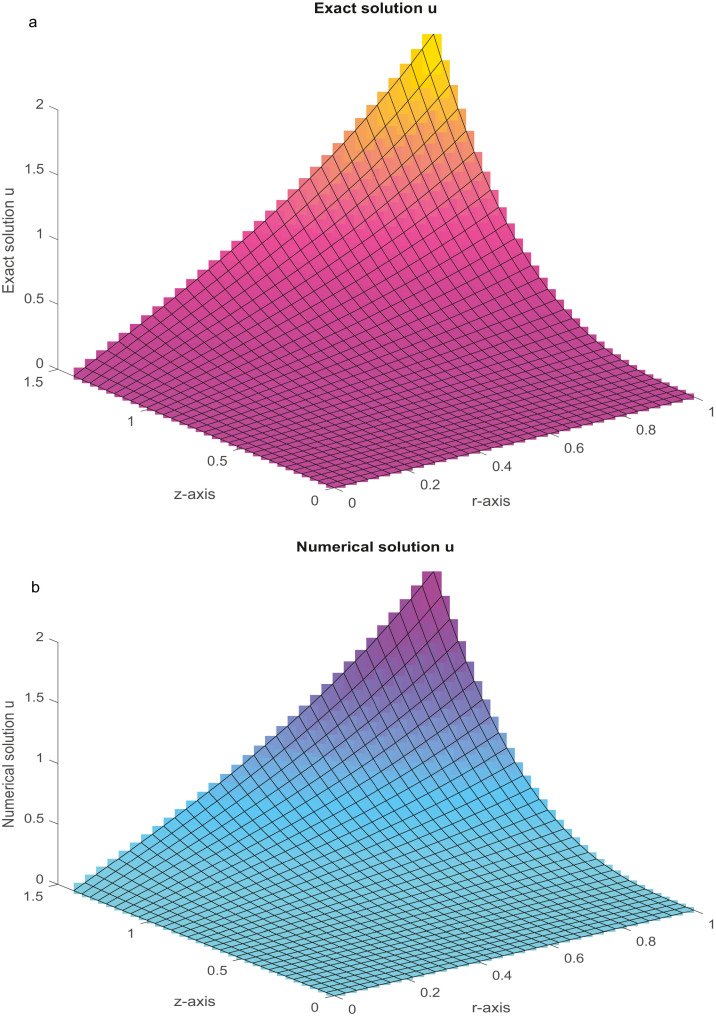

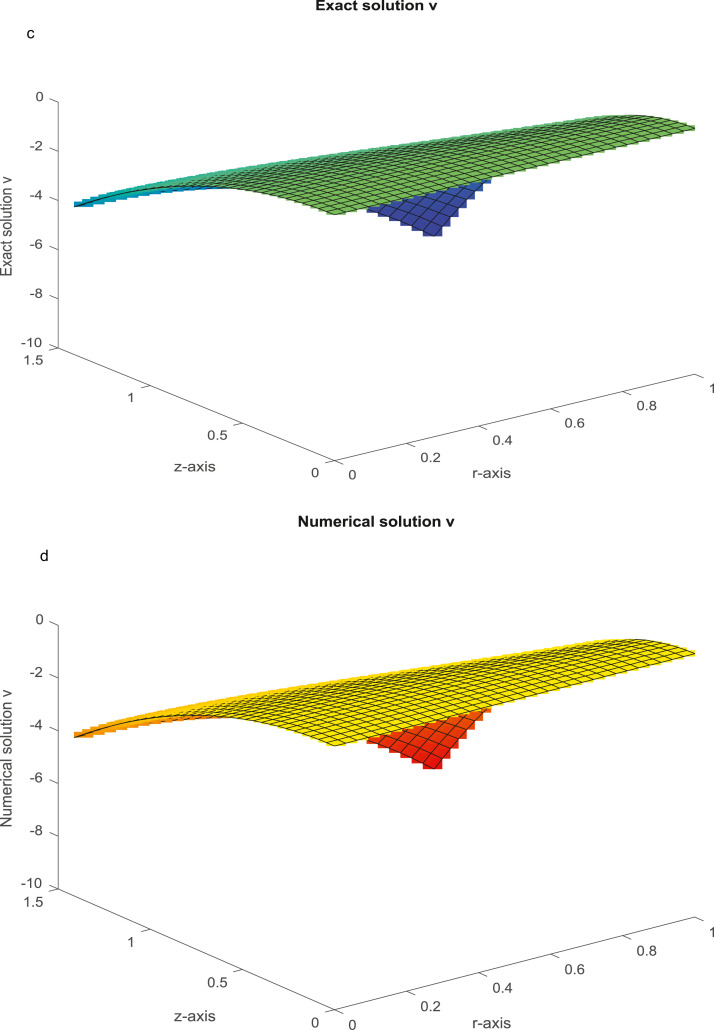
a: The graph of analytical solution, u, with *R_e_* = 10^3^ and *N_x_* = *N_y_* = 31. Fig. 5. b: The graph of numerical solution, u, with *R_e_* = 10^3^ and *N_x_* = *N_y_* = 31. Fig. 5. c: The graph of analytical solution, *v,* with *R_e_* = 10^3^ and *N_x_* = *N_y_* = 31. Fig. 5. d: The graph of numerical solution, *v*, with *R_e_* = 10^3^ and *N_x_* = *N_y_* = 31.

The analytical solutions are $u=r^{3}\;\text{sinhz},\;v=\;-4r^{2}coshz.$ The MAEs for the solutions *u* and *v* are given in Table 4, taking various values of the Reynold constant *R_e_*. The analytical and numerical plots for *u* and *v* are graphed in Figs. 5a-d for $N_{x}=N_{y}$=31 and $R_{e}=10^{3}.$

Example 5 (52) $$u_{xx}+\left(1+u^{2}\right)u_{yy}=u\left(u_{x}+u_{y}\right)+f\left(x,y\right),0<x<e,\;0<y<\frac{\pi }{2}.$$

**Table 4:** (Example 4): The MAEs.

$N_{x}=N_{y}$	Proposed FDM			Method [49]			Method [56]	
	$R_{e}=10$	$R_{e}=10^{2}$	$R_{e}=10^{3}$	$R_{e}=10$	$R_{e}=10^{2}$	$R_{e}=10^{3}$	$R_{e}=10$	$R_{e}=10^{2},10^{3}$
15 *u*	1.8317(−06)	2.0204(−05)	1.7178(−03)	7.6512(−06)	4.8900(−05)	3.0319(−03)	3.7991(−04)	Unstable
*v*	1.1949(−06)	1.6886(−05)	1.4376(−03)	4.7072(−06)	3.0462(−05)	2.9142(−03)	1.4492(−03)	
CPU time (sec)	(3.1818)	(4.4332)	(5.1043)					
31 *u*	1.1859(−07)	1.2679(−06)	1.3303(−04)	4.2316(−07)	2.9424(−06)	1.9105(−04)	9.1578(−05)	Unstable
*v*	8.2138(−08)	1.1302(−06)	8.9855(−05)	2.9392(−07)	1.9064(−06)	1.8073(−04)	3.5201(−04)	
CPU time (sec)	(8.4562)	(12.2167)	(15.1543)					
63 *u*	7.4638(−09)	7.9503(−08)	8.3304(−06)	2.6061(−08)	1.8196(−07)	1.1954(−05)	2.2508(−05)	Unstable
*v*	5.1803(−09)	7.0859(−08)	5.6823(−06)	1.8333(−08)	1.1844(−07)	1.1244(−05)	8.6797(−05)	
CPU time (sec)	(30.3634)	(41.4245)	(48.3198)

**Fig. 6:**
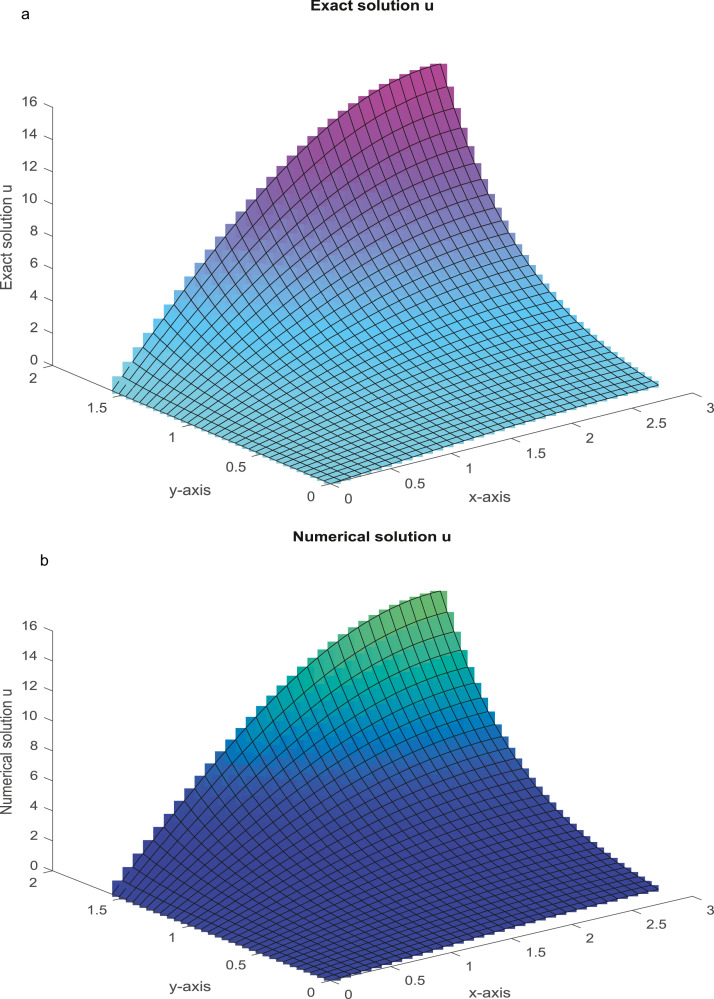
a: The graph of closed-form solution with *N_x_* *=* *N_y_* = 31. Fig. 6b: The graph of numerical solution with *N_x_* = *N_y_* = 31.

Clearly, this is a quasi-linear equation. The closed form solution is $u\left(x,y\right)=e^{x}\;\text{siny}.$ The MAEs are given in Table 5. The closed-form and numerical solutions are graphed in Figs. 6a and b, respectively, for $N_{x}=N_{y}$=31.

**Table 5:** (Example 5): The MAEs.

$N_{x}=N_{y}$	Proposed Method	Method [49]	Method [56]
15	1.3913(−05)	3.9473(−05)	3.5901(−04)
CPU time (sec)	(0.1818)		
31	8.6978(−07)	2.2858(−06)	9.1000(−05)
CPU time in (sec)	(0.8879)		
63	5.4196(−08)	1.4142(−07)	2.2652(−05)
CPU time in (sec)	(7.8873)

The order of the proposed method is $O(h_{x}^{4}+h_{x}^{2}h_{y}^{2}+h_{y}^{4})$. When $h_{y}\propto h_{x}$ or, $h_{x}\propto h_{y}$ the order of accuracy transforms as either $O(h_{y}^{4})$, or of $O(h_{x}^{4})$. In the given domain $\Omega_{h}$, $h_{x}=\gamma h_{y},\;\gamma \neq 1.$ The order of convergence can be computed using the formula

(53) $$\frac{\log\left(\frac{e_{h_{x1}}}{e_{hx_{2}}}\right)}{\log\left(\frac{h_{x1}}{h_{x2}}\right)},$$

where $e_{h_{x1}}$ and $e_{h_{x2}}$ refer to the MAEs of grid sizes $h_{x1}=\frac{x_{a}}{\left(1+N_{x1}\right)}\;$and $h_{x2}=\frac{x_{a}}{\left(1+N_{x2}\right)},$ respectively, with $h_{x1}\;\neq \;h_{x2}$. We have chosen, $N_{x1}=31$ and $N_{x2}=63.$ The order of convergence for all problems are computed in Table 6.

**Table 6:** (The rate of convergence): $h_{x1}=\frac{x_{a}}{32},\;h_{x2}=\frac{x_{a}}{64}$.

Example	Parameters, if any		Order of convergence
1			4.00
2	ε=0.1		3.99
	ε=0.01		3.98
	ε=0.001		3.98
3	$R_{e}=10$	foru	3.98
		forv	3.99
	$R_{e}=10^{2}$	foru	3.99
		forv	3.98
	$R_{e}=10^{4}$	foru	3.98
		forv	3.98
4	$R_{e}=10$	foru	3.98
		forv	3.98
	$R_{e}=10^{2}$	foru	3.99
		forv	3.99
	$R_{e}=10^{3}$	foru	3.99
		forv	3.98
5			4.00

## CRediT authorship contribution statement

**R.K. Mohanty:** Conceptualization, Funding acquisition, Investigation, Methodology, Project administration, Supervision, Writing – review & editing. **Nikita Setia:** Conceptualization, Data curation, Formal analysis, Funding acquisition, Investigation, Resources, Software, Validation, Visualization, Writing – original draft, Writing – review & editing. **Gunjan Khurana:** Conceptualization, Formal analysis, Funding acquisition, Resources, Software, Validation, Visualization, Writing – review & editing. **Geetan Manchanda:** Conceptualization, Formal analysis, Funding acquisition, Validation, Writing – review & editing.

## Declaration of Competing Interest

The authors declare that they have no known competing financial interests or personal relationships that could have appeared to influence the work reported in this paper.

## Data Availability

No data was used for the research described in the article.
